# Spatially resolved T cell receptor diversity mapping uncovers variability of the cancer immune microenvironment

**DOI:** 10.1016/j.ebiom.2026.106264

**Published:** 2026-04-24

**Authors:** Anastasia Magoulopoulou, Maria Chatzinikolaou, Andreas Metousis, Taobo Hu, Hui Yu, Ioannis Zerdes, Kang Wang, Theodoros Foukakis, Mengping Long, Karin Leandersson, Patrick Micke, Carina Strell, Mats Nilsson

**Affiliations:** a Science for Life Laboratory, Department of Biochemistry and Biophysics, Stockholm University, Stockholm, Sweden; b Department of Proteomics and Signal Transduction, Max Planck Institute of Biochemistry, Martinsried, Germany; c Department of Immunology, Genetics and Pathology, Uppsala University, Uppsala, Sweden; d Department of Oncology-Pathology, Karolinska Institutet, Stockholm, Sweden; e Theme Cancer, Karolinska Comprehensive Cancer Center and University Hospital, Stockholm, Sweden; f Breast Cancer Center, Theme Cancer, Karolinska University Hospital, Stockholm, Sweden; g Department of Translational Medicine, Cancer Immunology, Lund University, Malmö, Sweden; h Centre for Cancer Biomarkers CCBIO, Department of Clinical Medicine, University of Bergen, Bergen, Norway

**Keywords:** In situ sequencing, (ISS), T cell receptor, (TCR), Non-small cell lung cancer, (NSCLC), Spatially resolved transcriptomics, (SRT), TCR diversity, Breast cancer

## Abstract

**Background:**

T cell receptor (TCR) binding properties have been related to a wide range of pathological conditions, including infections, autoimmunity and cancer. Characterising the TCR repertoire is of great biomedical interest but it has been challenging due to its high structural diversity.

**Methods:**

In situ sequencing (ISS) is a suitable technique for spatial cell typing and linking gene patterns directly to specific histopathological features of large biopsy areas. We applied ISS through the commercial Xenium platform, with the addition of a custom panel specifically designed for TCR gene detection. Based on the IMGT database, we selected unique target sequences for TCR genes encoding the constant, variable and joining TCR chains. Additionally, we developed an analysis pipeline for the assignment of putative clonotypes based on simultaneous expression of alpha and beta variable TCR chains (TCRVβ/Vα pairs) at the single-cell level.

**Findings:**

Our approach captured specific immune cell distributions in relation to the individual sample clonality, as well as regional dominance of certain TCRVβ/Vα pairs in surgical non-small cell lung cancer (NSCLC) specimens and matching lymph node samples. Furthermore, we were able to study the spatiotemporal evolution of TCR repertoire on longitudinal FFPE biopsies from patients with breast cancer, during neoadjuvant treatment.

**Interpretation:**

This study highlights the implementation of target-based spatially resolved transcriptomics for the spatial characterisation of TCRVβ/Vα pairs at the single-cell level, without the need for prior sequencing. Our approach allows for spatial immune characterisation of diagnostic tissue samples with emphasis on T cell biology and accompanying T cell diversity.

**Funding:**

This study was supported from 10.13039/501100002794Cancerfonden (CAN 2021/1726), 10.13039/501100004359Swedish Research Council (Dnr: 2019-01238), U-CAN and the 10.13039/100016190Trond Mohn Foundation.

**Research in context**

**Evidence before this study**

T cells are responsible for identifying and acting against harmful foreign antigens, including cancer cells. The activation and subsequent clonal expansion of T-cell populations is essential for a strong immune response, and the characterisation, as well as the localisation of the TCR repertoire, is of great biomedical interest. However, this has been challenging because of the TCR repertoire's extensive structural diversity and the technical limitations of existing analytical methods.

**Added value of this study**

We introduce a TCR-focused in situ sequencing (ISS) approach using the Xenium platform, that targets TCR genes, achieving single-cell spatial mapping of T cell putative clones in preserved tissue. The putative clones were identified by the co-expression of specific alpha and beta chain transcripts (TCRVβ/Vα pairs). We also demonstrate applicability to non-small cell lung cancer and breast cancer tissue samples, capturing immune cell distributions, TCRVβ/Vα pair dominance, and spatiotemporal dynamics during treatment. The method combines spatial resolution, high sensitivity, and the ability to assess T cell states alongside TCR diversity.

**Implications of all the available evidence**

Our method enables a deeper understanding of how TCR clonality and immune cell localisation are associated with tumour progression, treatment response and immune evasion. Our findings support the potential of spatial TCR analysis for biomarker discovery in cancer diagnostics and monitoring, as well as allowing for patient stratification for immunotherapy.

## Introduction

**Fig. 1:**
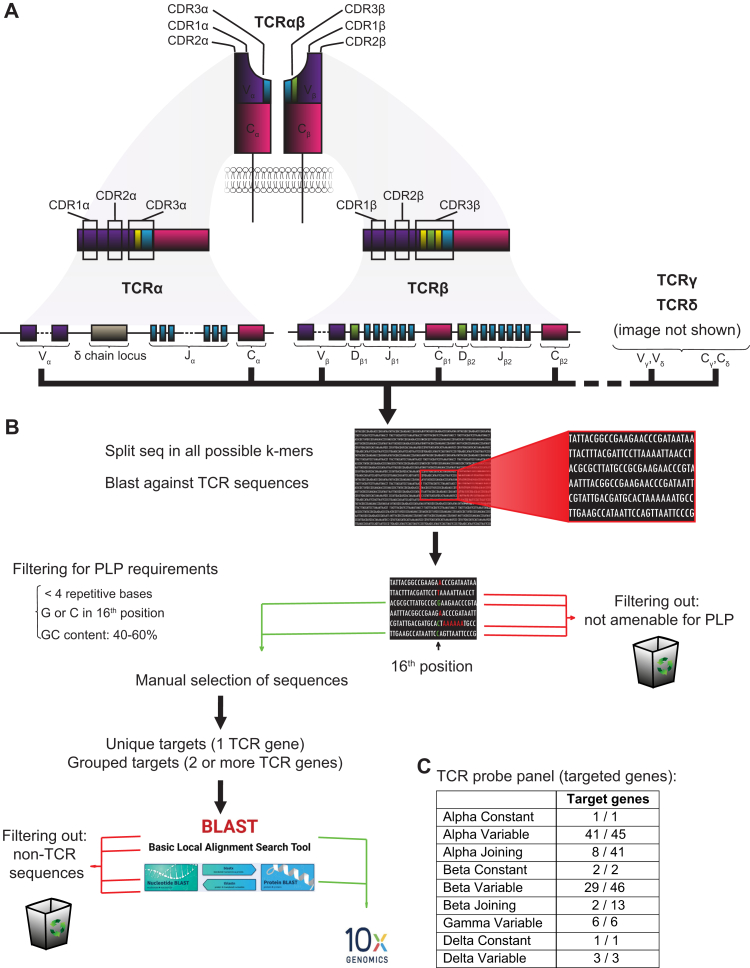
**Overview of design pipeline for T cell receptor variable genes.** A) Structure and generation of α/β T cell receptor. CDR1, CDR2 and CDR3 regions are color-coded. The position of the TCR genes on the receptor and on the chromosome locus is shown. B) Workflow for padlock probe target identification. Human TCR variable, joining and constant gene sequences for alpha, beta, gamma and delta chains were retrieved and split in all possible 30-mers. Uniqueness and cross-reactivity for each 30-mer was tested using BLAST against all other TCR 30-mers. Selected 30-mers were filtered to match padlock probe design criteria: less than four repetitive bases, G or C in the 16th position and G-C content between 40 and 60%. Filtered 30-mers were further assessed for specificity by BLAST against the human transcriptome. Probe backbone sequences were added to the selected 30-mers by 10× Genomics. C). Number of TCR gene targets included in the panel. Probe coverage is reported as the number of genes targeted, relative to the total number of known genes.

T cells are a key component of the adaptive immune response, playing a crucial role in recognising and responding to foreign antigens. They exert their function through the T cell receptor (TCR), a specialised protein heterodimer on the T cell surface, that recognises antigenic peptides presented by other cells via their Major Histocompatibility Complex (MHC) molecule I or II. In human peripheral blood, the majority of T cells express TCRs composed of one alpha and one beta chain (TCR αβ), whereas less than 5% of all T cells express TCRs composed of gamma and delta chains (TCR γδ).^1^ The vast TCR diversity is primarily the result of the somatic recombination of Variable (V), Diversity (D) (only for beta and delta chains), Joining (J) and Constant (C) TCR gene segments, during T cell development in the thymus.^2^ Additional diversity of the TCR is achieved by the complementary determining regions 1, 2, and 3 (CDR1, CDR2, CDR3). The CDRs 1 and 2 are entirely encoded by germline DNA segments, whereas CDR3 is the most variable one, since it spans the V(D)J junction regions where random nucleotide additions and/or deletions take place during recombination^3^, ^4^, ^5^ (Fig. 1A). Mathematically, TCR diversity has the potential to create 10^18^ distinct TCR proteins.^6^ However, in an individual human, the actual TCR repertoire is estimated to range between approximately 10^8^–10^9^ distinct TCRs.^4^ This TCR diversity enables the immune system to recognise any antigen and initiate immune responses against it.

Tumour infiltrating lymphocytes are a defining factor of the tumour microenvironment and influence the induction of a successful anti-tumour immune response.^7^ During recent years, studies have focused on investigating how the TCR repertoire of solid tumours, their surrounding tissue as well as the peripheral blood is associated with disease progression and therapy efficacy, particularly immunotherapy. In metastatic triple-negative breast cancer, studies have shown that TCR repertoires gradually lose diversity with cancer progression, leading to convergent evolution into similar clonotypes, accompanied by the emergence of tumour immune evasion mechanisms, while peripheral blood T cells retained some cytotoxic potential.^8^ In NSCLC, post-treatment analysis of neoadjuvant immunotherapy revealed that patients with major pathologic responses had tumour beds enriched with T-cell clones that expanded in the periphery during treatment.^9^ Yet as the impact of TCR repertoire on disease outcomes appears to vary between cancer types,^4^ detailed analyses of TCR diversity are crucial for understanding cancer progression and optimising treatment strategies. Furthermore, the spatial localisation of T-cell clones, including whether they reside intratumourally, peritumourally, or within tertiary lymphoid structures, may give clinically relevant information.^10^

TCR repertoire analysis, however, is challenging due to the high diversity of TCRs and their typically low transcript abundance in tissue samples.^11^^,^^12^ Many methods have been applied throughout the years to assess the TCR repertoire in tumours, each with its own limitations. Flow cytometry is restricted by the availability of specific antibodies and requires freshly isolated cells. Multiplex PCR, although widely used, can introduce amplification bias. Bulk sequencing methods, especially DNA-based ones–although cost-effective–do not account for allelic exclusion, thus risking an overestimation of TCR diversity, and (along with the RNA sequencing methods) the dependency on amplicon length limits their capacity to fully capture TCRs. Also, they have to rely on computational prediction for alpha and beta chain pairing. Even the use of 5′ RACE for capturing of the full CDR3 region remains limited by a suboptimal adaptor ligation step, which reduces capturing efficiency.^5^^,^^13^^,^^14^ Single-cell RNA sequencing with its latest progress on 5’ sequencing adaptation for V(D)J capture overcomes the issue of pairing alpha and beta chains but lacks spatial information. In the emerging spatially resolved transcriptomics field, recent advances allow for spatial capture of TCR sequences, however the relatively low sensitivity and computational pairing of alpha and beta chains still need to be addressed.^11^^,^^12^^,^^15^^,^^16^ Spatial information on clonal T cell expansion within tumour tissue is yet highly desirable, as it could provide valuable insights and reveal differential effects on distinct T cell populations, such as intratumoural versus excluded T cells. However, current spatial methods often lack true single-cell resolution and exhibit low detection sensitivity, limiting simultaneous assessment of clonal identity and T cell state, since many relevant genes beyond the TCRs are poorly captured. In addition, standard targeted technologies have not been able to recover V gene usage due to the absence of targeted probes for these highly diverse regions.

In this study, we adapted in situ sequencing^17^, ^18^, ^19^ using the Xenium platform to spatially map TCR gene sequences in diagnostic tissue samples from non-small cell lung cancer (NSCLC) and breast cancer. By designing a complementary TCR probe panel, we identified TCR alpha (TRAV) and beta (TRBV) variable gene pairings (TCRVβ/Vα pairs). CD8 T cells showed greater TCRVβ/Vα expansion than activated or tertiary lymphoid structure–associated CD4 T cells, with dominant CD8 clones localised near cancer cells. In longitudinal breast cancer biopsies during neoadjuvant therapy, non-responders exhibited reduced T-cell diversity over time. Overall, our approach provides a highly flexible and valuable tool for high-throughput spatial assessment of TCR diversity in combination with transcriptomic profiling at single cell resolution.

## Methods

### Ethics

#### Tissue material from patients with NSCLC

This study includes sections from surgical specimens from 2 patients with NSCLC from the previously described Uppsala II cohort, for whom RNA-seq and Lymphotrack data was available.^20^^,^^21^ Both patients were female and sex was not considered as a biological variable in downstream analyses. This study was conducted according to the Declaration of Helsinki and was approved by the Ethical Review Board Dnr 2012/532 including a waiver of written informed consent. Individual patient characteristics are listed in Table S1.

#### Tissue material from patients with breast cancer

Formalin-fixed paraffin-embedded (FFPE) core needle biopsies have been obtained from six female patients diagnosed with early-stage oestrogen receptor-positive/human epidermal growth factor receptor 2-negative (ER+/HER2−) invasive breast cancer who received 12 weeks of neoadjuvant treatment with the cyclin-dependent kinase 4 and 6 (CDK4/6) inhibitor palbociclib in combination with endocrine therapy, within the randomised phase II PREDIX LumB clinical trial (NCT02603679).^22^^,^^23^ Paired biopsies were collected at baseline (treatment-naive) and on-treatment (after 12 weeks). Objective radiologic response (ORR_12_) was evaluated at 12 weeks, classifying patients into responders (R) and non-responders (NR). None of the patients achieved a pathological complete response at 12 weeks. The clinical trials and all the correlative analyses have been approved by the Regional Ethical Committee in Stockholm and the Swedish Medical Product Agency (PREDIX LumB trial: Dnr 2014/1492-31/4). All patients have provided written informed consent. Individual patient characteristics ate listed in Table S2.

### Probe selection and design

To design In Situ Sequencing Padlock Probes (PLPs) that can specifically differentiate between TCR chain genes of the same gene group, we developed a pattern matching-based R pipeline. The input in this R script is TR exon (DNA) transcript sequences in FASTA file format. The exon transcript sequences for all the known human constant and variable TCR α, β, γ and δ genes (excluding pseudogenes) were retrieved from the IMGT Repertoire (Ig and TR) database (https://www.imgt.org/IMGTrepertoire/) (Fig. 1A). For details see Supplementary Material and Methods.

### Xenium in situ gene expression

Fresh frozen and FFPE 10 μm tissue sections were placed on Xenium slides and processed according to the manufacturer's instructions with Predesigned Human Multi-Tissue and Cancer panel (377 genes) and custom TCR gene panel (98 genes). For details see Supplementary Material and Methods and Fig. S1. Xenium slides were then loaded for imaging and analysis on the Xenium Analyser instrument following Decoding and Imaging user guide (User guide CG000584). Instrument software (versions 1.8.2.1 and 1.7.6.0) and analysis software (version 1.7.1.0) were used. No de novo sequencing data were generated, consequently no raw sequencing data or accession numbers are applicable to these experiments.

### Xenium datasets

The datasets included in this study were formatted in the anndata format using a previously published function^24^ (https://github.com/Moldia/Xenium_benchmarking). Nuclei segmentation of the identified cells was selected. For the samples deriving from the PREDIX LumB clinical trial, cell segmentation was based on fluorescent markers for membrane boundaries, 18s RNA and interior proteins. The transcripts were assigned to cells and the annotated data objects were created for each sample.

### De novo clustering

For the downstream analysis, cells with a minimum expression of at least 5 transcripts and 3 genes were selected. The gene expression of the cells was normalised and transformed to be clustered with the Leiden algorithm. Neighbourhood graphs were computed considering 15 principal components and 10 neighbours. The clusters were annotated based on the top 15 differentially expressed genes per cluster, using well-established markers and reference datasets from CZ Cell X Gene database (https://cellxgene.cziscience.com/).

For the clustering and cell annotation of samples deriving from the PREDIX LumB clinical trial, we performed label transfer from the annotated scRNA-seq dataset^25^ onto our Xenium spatial data using Scanpy (version 1.10.4) in Python (version 3.11.3) via its ‘ingest’ functionality. For details see Supplementary Material and Methods.

### TCR density and diversity binned plots

The tissue area was binned in hexagons with a grid size of 300 pixels. Based on the transcript content of each bin, the total number of TCR genes per hexagon was calculated to build a density graph of gene expression. For the diversity plots, we calculated the fraction of expression of each TCR variable gene by the total counts of all the TCR variable genes per hexagon. The fraction (percentage) of expression of the most expressed TCR variable gene in a bin is displayed as max fraction per bin.

### TCR clonotyping

For assigning putative clonotypes (TCRVβ/Vα pairs) to single cells, the AnnData object was parsed and its spatial coordinates (x and y) were added to the obsm layers “spatial” and “xy_loc” which were used to make the spatial plots. Then, the distributions of counts/genes per cell and cells (positions) per gene were plotted. Cells with a minimum of 5 counts per cell and genes with a minimum of 3 detected cells per gene, were kept.

The filtered AnnData was used to compute clonotype IDs and types for each cell. The procedure iterated all the cells and computed a clonotype type as follow:

• orphan_beta if the number of alpha genes is 0 and the number of beta genes is 1
• orphan_alpha if the number of alpha genes is 1 and the number of beta genes is 0
• ab_single_pair if the number of alpha genes is 1 and the number of beta genes is 1
• ab_multi_chain if the number of alpha genes is 2 and the number of beta genes is 2
• extra_alpha if the number of alpha genes is 2 and the number of beta genes is 0 or 1
• extra_beta if the number of alpha genes is 0 or 1 and the number of beta genes is 2
• ab_unknown the remaining cases

The respective gamma/delta types were computed in the same way but using the gamma and delta genes instead. Only the ab_single_pair and gd_single_pair putative clonotypes were used for further analysis. All code for the TCR clonotyping is available online at https://github.com/jfnavarro/ISS_TCR.

### Neighbourhood analysis

Spatial proximity and neighbourhood enrichment were assessed using permutation-based tests on spatial neighbour graphs constructed with Squidpy^26^ (https://github.com/scverse/squidpy) by selecting the neighbouring cells within a 125-pixel radius for each cell, which approximately corresponds to a two-cell diameter. For more details, see Supplementary Materials and Methods.

### Neighbourhood clustering

BANKSY algorithm^27^ (https://github.com/prabhakarlab/Banksy) was used for incorporating neighbourhood information for clustering. The features of each cell were taken into account together with an average of the features of its spatial neighbours and with neighbourhood feature gradients.

### Statistics

#### Gini index

The Gini Index (Gini coefficient) was calculated to assess the inequality in TRV transcript counts among cells in clusters.

### Jaccard Similarity Index

The abundance-based Jaccard Similarity Index (J_abd_) was calculated to highlight the overlap of TCRVβ/Vα pairs in biopsies from patients with breast cancer between two time points (baseline and on-treatment).

For details on their calculation see Supplementary Material and Methods.

### Role of funders

The funders of the study did not have any role in study design, data collection, data analysis, interpretation, or writing of the report.

## Results

### T cell receptor gene panel design

For the padlock probes (PLPs) design we identified target sequences against the constant and variable genes that code for the α, β, γ, and δ chains of the T-cell Receptor (TCR) as described in Methods (Fig. 1A and B). These genes are known to be well conserved, with a high percentage of sequence identity. The final target sequences were used by 10× Genomics to create a customised TCR gene panel, which includes probes against 1 α, 2 grouped β, and 1 δ constant gene, as well as, 41 unique (39 grouped) α variable genes, 8 α joining genes, 33 unique (29 grouped) β variable, 2 β joining, 6 unique (4 grouped) γ variable, and 3 δ variable genes (Fig. 1C, Tables S3 and S4). This panel was combined with the commercial 10× Xenium pan-tissue panel, which targets an additional 377 genes covering immune, cancer, and stromal markers (Table S5) (Fig. 1B).

### Spatial distribution of TCRs in relation to T cell subsets in NSCLC

Our initial aim was to identify TCR distribution patterns and their spatial relationship to T cell subsets in NSCLC tissue. For this purpose, we analysed one adenocarcinoma (AC) case and one squamous cell lung carcinoma (SqCC) case of known clonality,^21^ along with their corresponding, unaffected lymph nodes. We cross-validated TRBV gene detection using multiple approaches, including Lymphotrack, RNA-seq, ISS, and Xenium (Table S6). The overall repertoires were largely consistent and reflected the individual sample characteristics. Differences between methods are expected due to biological heterogeneity and sampling effects, as well as method-specific target design, sensitivity and detection limits. Nevertheless, highly expressed and expanded TCR genes are consistently detected across platforms, supporting the robustness of the observed dominantly expressed genes, which was the case for less diverse AC sample (Gini Index = 0.65). The SqCC sample was highly diverse (Gini Index = 0.14)^21^ therefore the restricted agreement between methods reflects the sample's biology (Tables S6–S8).

**Fig. 2:**
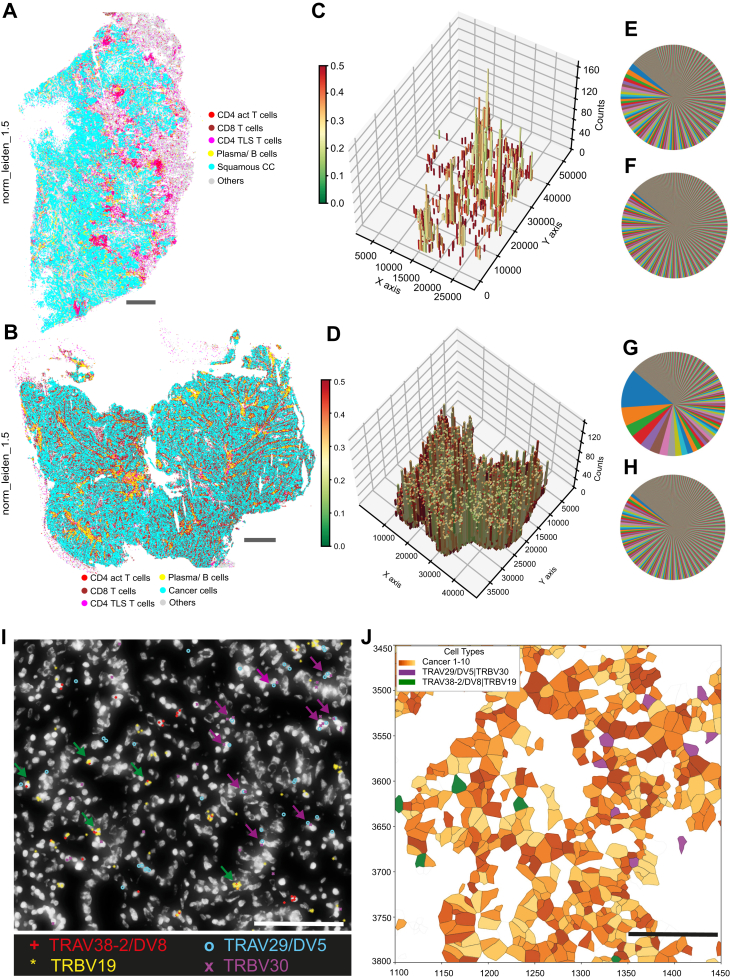
**Spatial distribution T cells and T cell receptor genes in SqCC and AC human biopsies.** A and B): Spatial maps depicting the distribution of CD4 activated T cells, CD8 T cells, plasma cells and B cells in relation with the identified cancer populations in SqCC (A) and AC (B). Scale bar = 1000 μm. C and D): 3D plot depicting the density of TRBV genes as the number of counts per bin (z axis) and diversity depicted as the fraction percentage of the dominant TRBV gene in the bin (maximum 50%) for SqCC (C) and AC (D). X and Y axis indicate number of pixels in the image (5000 pixels = 1000 μm). E, F, G and H): Relative frequency and proportion of identified TCRVβ/Vα pairs in single cells in primary tumours (E and G) and healthy lymph nodes from the same patients (F and H). I) Presence of TRAV38-2/DV8, TRBV19, TRAV29/DV5 and TRBV30 genes in AC biopsy. Green arrow showing single cells with TRAV38-2/DV8|TRBV19 combination (TCRVβ/Vα pair) and blue arrows showing single cells with TRAV29/DV5|TRBV30 combination. Scale bar = 100 μm. J) Spatial plot of (I) showing TRAV29/DV5|TRBV30 (purple) and TRAV38-2/DV8|TRBV19 (green) T cells and cancer populations. Scale bar = 100 μm.

Cell typing after Leiden clustering revealed 36 cellular subtypes across both tumour samples (Fig. S2A). Notably, the immune cell profiles differed between the two tumours, with the AC showing a strong prevalence of CD8 T cells (Sup. Fig. S2B and C), while in the SqCC sample, activated CD4 T cells and tertiary lymphoid structure (TLS) associated CD4 T cells were more abundant (Fig. S2B and C). Histologically, the SqCC case showed pronounced compartmentalisation between cancer and stroma regions, as typical of this subtype; CD4 and TLS associated CD4 T cells localised in the stromal compartment (Fig. 2A). In contrast, the AC case, showed high infiltration of CD8 T cells in the tumour compartment, while CD4 and TLS associated CD4 T cells located at the tumour-stroma edge in close proximity with plasma/B cells (Fig. 2B).

In order to first outline the TCR distribution and confirm the simultaneous presence of both alpha and beta V genes in the same tissue areas, we used a binning approach in which the full tissue area was split into hexagons of 300-pixel size (roughly containing 5 cells per hexagon) (Figs. S3 and S4). In each bin, the density and diversity of the TCR alpha and beta variable genes were assessed. As expected, the hexagons containing TCRV counts closely mirrored the distribution patterns of T cells within the corresponding tissues (for SqCC: Fig. 2C, Fig. S3A and B; for AC: Fig. 2D, Fig. S4A and B). As a measure of TCR diversity, we calculated the fraction (percentage) of each TRBV or TRAV gene to the total number of TRBV or TRAV gene counts in each hexagon and plotted the maximum value (Fig. 2C and D, Figs. S3C and D, S4C and D). We defined a maximum fraction of 50% (or more) for one TCRV gene to indicate low diversity. Hexagons with less than 10 total TRBV or TRAV counts were filtered out to not falsely indicate low diversity due to low count numbers. In SqCC, regions abundant in CD4 and TLS associated CD4 T cells exhibited higher TCRV diversity as compared to the CD8 cell-rich hexagons, which were located closer to the cancer compartment and showed lower TCRV diversity (Fig. 2A and C). In the AC, which is characterised by predominant CD8 infiltration, displayed overall a lower TCR diversity. Generally, both TRBV and TRAV genes followed similar distribution patterns, though TRAV genes demonstrated higher diversity across both histological subtypes (Figs. S3C and D, S4C and D).

### Dominant TCRVβ/Vα pairs are located proximal to tumour cells

As a next step, we were interested in assigning putative clonotypes to single T cells. To achieve this, we developed the TCR clonotyping pipeline (see Methods) for investigating the TCR gene expression at single-cell level. This pipeline extracts cells expressing, one TRAV and one TRBV gene (TCRVβ/Vα pair), apart from other genes. Using the same approach, we could extract cells that express one gamma variable and one delta variable. We identified unique combinations of TRAV and TRBV genes as putative clonotypes, to which we refer to as TCRVβ/Vα pairs with likely similar binding properties to antigens. TCRVβ/Vα pairs were primarily detected in T cell populations, whereas non-T cell types showed minimal background signal, consistent with the specificity of the assay (Fig. S5A). Additionally, TCRVβ/Vα pairs were the predominantly detected clonotype type (see Methods) and were the only category considered for downstream analyses (Fig. S5B).

We assessed the number of unique TCRVβ/Vα pairs in AC and SqCC biopsies, as well as the corresponding unaffected lymph nodes. The total TCRVβ/Vα pair counts were similar across for the two samples with 681 detected TCRVβ/Vα pairs identified in SqCC and 688 in AC. In contrast, the lymph nodes demonstrated higher diversity, with 1164 TCRVβ/Vα pairs for the SqCC-matching lymph node and 1153 for AC-matching lymph node. This finding underscores the greater T-cell diversity in the lymphoid tissue. Moreover, the most dominant TCRVβ/Vα pairs within the lymph nodes accounted for 0.64% of the total pairs in the SqCC (TRAV4|TRBV20-1) -matched lymph node and 0.94% in the AC (TRAV26-1|TRBV20-1). However, within the primary tumours the most dominant TCRVβ/Vα pair (SqCC: TRAV26-1|TRBV20-1, AC: TRAV29/DV5|TRBV30) accounted for 2.1% in SqCC and 12.2% in AC, highlighting the selectivity of TCRs in the tumour site (Fig. 2E–H). Notably, the most dominant TCRVβ/Vα pair in the AC primary tumour was absent among the top 400 TCRVβ/Vα pairs in the matching lymph node.

**Fig. 3:**
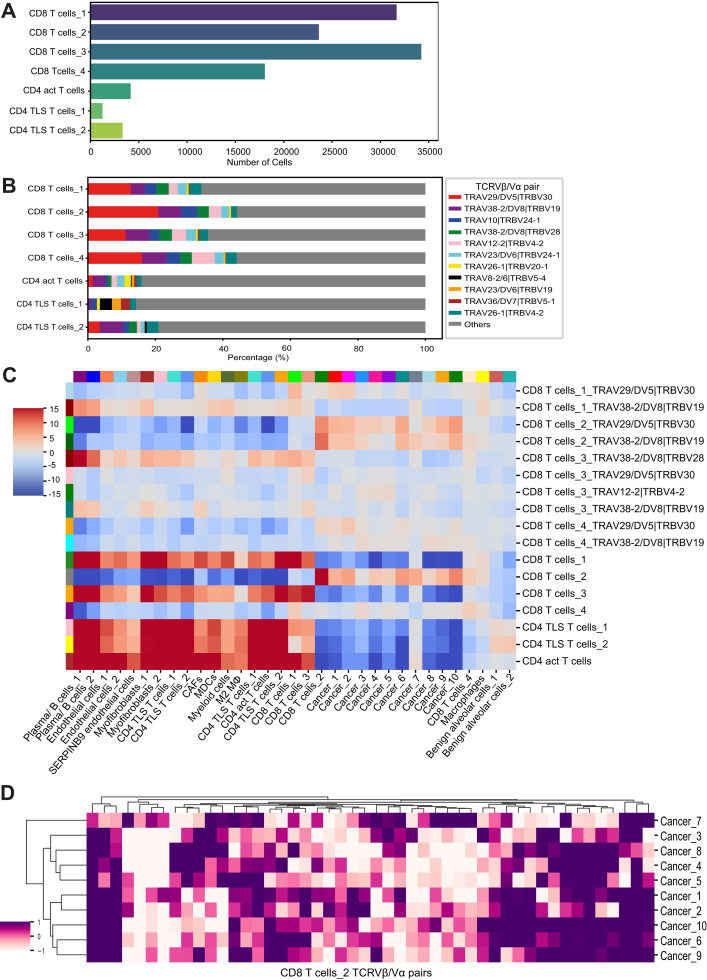
**T cell populations and TCRVβ/Vα paired cell distribution in adenocarcinoma.** A) Number of cells of identified T cell subtypes. B) Proportions of identified TCRVβ/Vα pairs in T cell subsets. The three most abundant TCRVβ/Vα pairsof each T cell subtype are colour-coded and the rest of the TCRVβ/Vα pairs are represented as Others. C) Neighbourhood analysis of the identified T cell subtypes and their most abundant TCRVβ/Vα pairs in relation with the rest cell types on the tissue within a 125-pixel radius. D) Neighbourhood analysis of all the identified TCRVβ/Vα pairs of CD8 T cells_2 subtype in relation with the cancer populations within a 5 cell radius. TCRVβ/Vα pairs with more than 5 cells are taken into account.

We next explored the location of dominant TCRVβ/Vα pairs in the AC tissue (Fig. 2I and J) and neighbourhood analysis revealed that the dominant T cells were proximal to cancer cells (Fig. 3C).

### T cell subsets and their TCRVβ/Vα pair organisation

We also identified the T cell subpopulations found in AC and SqCC. The AC biopsy, which was generally enriched for CD8 T cells, showed 4 different CD8 subtypes (Fig. 3A), which shared similar gene profiles. However, CD8 T cells_1 expressed the activation marker *CYTIP*, CD8 T cells_2 expressed *CFB*, a marker for alternative complement activation, CD8 T cells_3 showed an intermediate profile with simultaneous expression of activation (*GRZA*, *GRZB*) and exhaustion markers (*LAG3*), and CD8 T cells_4 expressed less *GRZB* and more *LAG3* (Table S9). Activated CD4 T cells and two subsets of TLS associated CD4 T cells were less abundant than CD8 T cells in AC (Fig. 3A). The most dominant detected TCRVβ/Vα pairs were shared and commonly detected across all the CD8 T cell subsets (Fig. 3B). TLS associated CD4 T cells_2 also shared few TCRVβ/Vα pairs with CD8 T cells but displayed overall a higher diversity (Fig. 3B).

We investigated which cell types were enriched in the local neighbourhoods of T cell subpopulations and the most dominant TCRVβ/Vα pairs. CD8 T cells of subtype 2 (positive for CFB expression) and CD8 T cells_4 (potentially exhausted T cells) were closer to the cancer cells in the AC sample, than the other CD8 subtypes. The most dominant TCRVβ/Vα pairs within these CD8 cell subsets followed the same spatial pattern. Interestingly, the most abundant CD8 T cell subset 1, with the dominant TCRVβ/Vα pair TRAV29/DV5|TRBV30, was located in close proximity to cancer cell subsets 1 and 2, both of which exhibited a high proliferation profile compared to the other cancer clusters (Cancer 3–10) (Fig. 3C).

Activated CD4 T cells, TLS associated CD4 T cells and CD8 T cells of subtype 1 (*CYTIP* expression) and 3 (activation and exhaustion phenotype), were located farther from the cancer cells but closer to other immune and stromal cells.

To assess whether all the TCRVβ/Vα pairs of a particular T cell subtype, and not just the dominant TCRVβ/Vα pair, associate within similar cellular neighbourhoods, we performed neighbourhood analysis for the cancer cell subpopulations in relation to all TCRVβ/Vα pairs in CD8 T cells_2 subset. The detected TCRVβ/Vα pairs exhibited varying proximity patterns to the different cancer cell subpopulations (Fig. 3D), which suggests antigen binding site versatility among the TCRVβ/Vα pairs of a single T cell subset (Fig. 3D).

Similar trends were observed in the SqCC sample, which was enriched for activated CD4 T cells as well as TLS associated CD4 T cells (Fig. S2C, Fig. S6A), which also comprised the majority of identified TCRVβ/Vα pairs (Fig. S6B). Similar to the AC case, activated CD4 T cells and TLS associated CD4 T cells were close to other immune and stromal cells (Fig. S6C). There was no proximity of CD8 T cells to SqCC cancer cells, reflecting the low degree of CD8 T cell infiltration in the biopsy (Fig. S6C). CD4 T cell subpopulations had shared TCRVβ/Vα pairs and maintained a high diversity (Fig. S6D), while CD8 T cell subsets showed less diversity and had distinct TCRVβ/Vα pairs compared to CD4 T cells.

### Characterisation of T cell-rich spatial domains

**Fig. 4:**
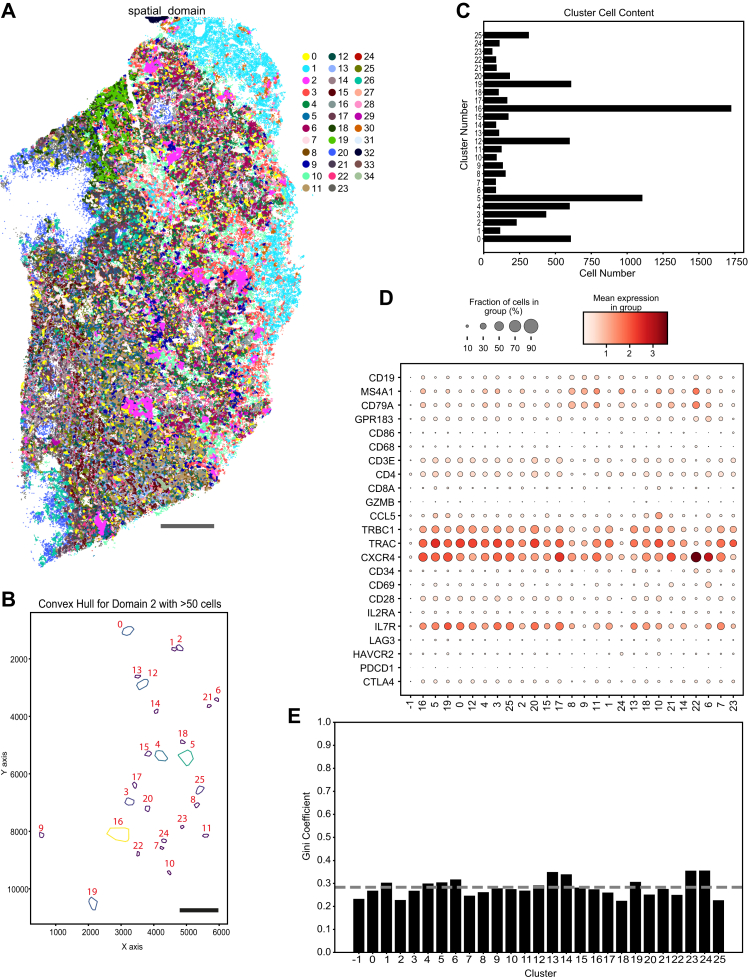
**Niche analysis for T cell populations.** A) Spatial domain identification. Scale bar = 1000 μm. B) Identification of CD4/CD4 TLS associated T cells spatial domains sharing the same microenvironment (minimum neighbourhood content = 50 cells). Scale bar = 1000 μm. C) Number of cells per identified neighbourhood cluster. (Cluster −1 = area that doesn't belong to CD4/CD4 TLS associated T cells neighbourhood). D) Relative expression of selected transcripts in the neighbourhood clusters. E) Gini Index per neighbourhood cluster. Average of neighbourhood clusters 0–25 indicated as dotted line.

Given the limited lymphocyte infiltration and the CD4 rich aggregates in SqCC (Fig. 2A), we used the BANKSY algorithm to identify spatial domains. The algorithm takes into consideration a cell's own gene expression as well as the expression in its neighbourhood together with the magnitude of expression in a set gradient. We identified Spatial domain 2, spanning across 25 regions, that consists mainly of activated CD4/TLS associated CD4 T cells, a composition that resembles TLS-like structures (Fig. 4A, B and C, Fig. S7).

The regions of Spatial Domain 2 were enriched for CD4 T cell markers (TRBC1, TRAC, CD3, CD4, CXCR4) and moderately enriched for B- and plasma cell markers (CD19, MS4A1, CD79A). Expression of dendritic cell and macrophage markers (GPR183, CD86, CD68) as well as CD8a and early T cell activation and exhaustion markers (CCL5, GZMB, LAG3, HAVCR2, PDCD1, CTLA4) was low (Fig. 4D. Additionally, the average Gini Index was 0.28 indicating high diversity of TCR genes in these clusters (Fig. 4E). These findings suggest formation of immature TLS-like structures.

### Spatiotemporal monitoring of TCR repertoire in breast cancer

**Fig. 5:**
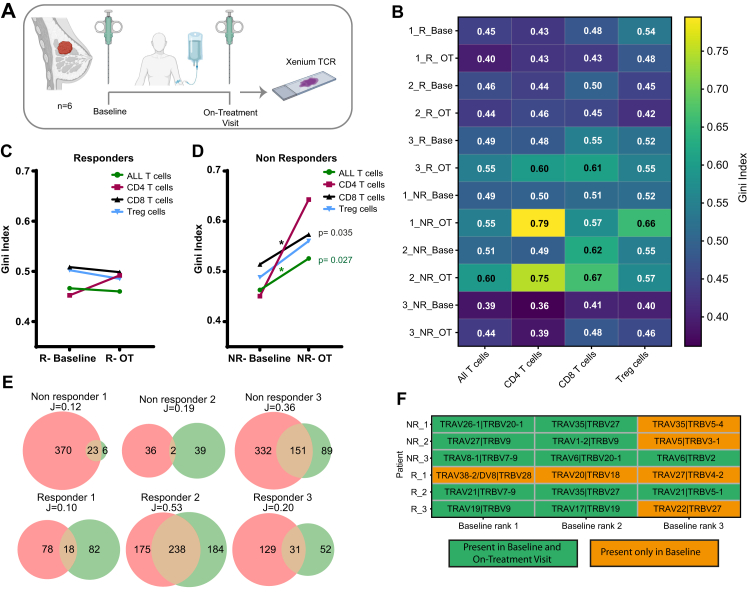
**TCR clonality and its temporal evolution in tumours from patients with early breast cancer.** A) Experimental set up for biopsies from six patients with breast cancer (n = 6) with ER-positive/HER2-negative disease, enrolled in the neoadjuvant PREDIX Lum B clinical trial (NCT02603679) (image made with BioRender). B) Gini Index score heat map per patient (NR: Non-responder, R: Responder), time point [baseline or on-treatment (after 12 weeks of treatment)] and T cell subset (cut-off = 15 cells per subset). Gini Index change for each T cell subset between baseline and on-treatment (OT) time points (paired 2-tailed student t-test, n = 3). C) Responders group and D) Non-responders group (All T cells p-value = 0,035, CD8 T cells p-value: 0,027). E) Venn diagrams of TCRVβ/Vα pairs that are present in baseline (pink circle), on-treatment (green circle) or both (intersection) time points in the longitudinal biopsies from breast cancer patients. Jaccard Similarity Index indicates dissimilar (close to 0) to identical (close to 1) TCRVβ/Vα pair compositions. F) Table indicating whether the top-3 most abundant TCRVβ/Vα pairs in baseline biopsies for each patient (NR: Non-Responder, R: Responder) are also present (green) or absent (orange) in the on-treatment biopsies.

We were also able to identify TCRVβ/Vα pairs in FFPE needle biopsies taken at the time of diagnosis (baseline) and after 12 weeks of neoadjuvant and endocrine treatment (on-treatment) from six patients with ER+/HER2-early breast cancer. The patients belonged either to the Responder or Non-responder group (n = 3 each) (Fig. 5A). We observed that the diversity of T cell receptor based on Gini Index differs between baseline and on-treatment time points as well as between T cell subtypes (Fig. 5B). Non-responders show decreased TCR diversity on-treatment, mainly driven by CD8 T cells, whereas in responders, no significant changes were observed (Fig. 5C and D), suggesting potential expansion of non-functional or exhausted T cells through the course of treatment in non-responders. Additionally, we observed that even if the overlap between maintained TCRVβ/Vα pairs between baseline and on-treatment time points was variable (Jaccard Similarity Index 0.1–0.53) (Fig. 5E), the most abundant TCRVβ/Vα pairs that were found at baseline they were maintained on-treatment in five out of the six patients (Fig. 5F).

## Discussion

In this proof-of-concept study, we developed a targeted, padlock probe- and image-based approach to study the spatial TCR repertoire without the requirement for prior TCR sequencing. Our approach allows for single-cell resolution and high throughput, while at the same time maintains the histopathological information.

We analysed the two samples representing the major NSCLC subtypes, squamous cell lung carcinoma (SqCC) and lung adenocarcinoma (AC), which showed distinct TCR gene distribution patterns based on their specific histopathology. Across the samples, T cell identity was confirmed by co-localisation of constant and variable TCR alpha and beta genes in single cells, and we observed higher TRAV than TRBV diversity, consistent with prior bulk TCR analyses.^28^

In the AC sample we observed high immune infiltration with high CD8 T cell presence and TCRVβ/Vα expansion. We observed that CD8 T cells had the most expanded TCRVβ/Vα populations, confirming previous literature emphasising that tumoural T cell clonality is primarily driven by CD8 T cell proliferation.^29^^,^^30^ The most abundant TCRVβ/Vα pairs of CD8 T cells (Fig. 3), were predominantly localised close to cancer cells, which aligns with previous observations,^21^ and is consistent with their cytotoxic function. In contrast, CD4 T cells, which exhibit a broader functional diversity -including activation of M1 macrophages and B cells-showed a more spatially distributed pattern.

In contrast, the SqCC sample showed low CD8 T cell infiltration but high presence of activated and TLS associated CD4 T cells. These CD4 T cells were primarily organised in TLS-like structures rather than infiltrating tumour nests. We were able to identify and characterise spatial domains enriched in mixed populations of activated and TLS associated CD4 T cells, the latter likely representing T helper cells. Plasma cells, B cells and myeloid dendritic cells (MDCs) were also present, suggesting immature TLSs.^31^^,^^32^ Gini Index of these domains indicated highly diverse TCRs, in line with previous descriptions of TCRs in TLS-like regions.^33^

We also observed increased TCRVβ/Vα expansion at tumour sites compared to matched unaffected lymph nodes and healthy tissues, which showed greater diversity. This likely reflects broader immune surveillance outside the tumour,^34^ whereas the tumour microenvironment promotes clonal expansion and repertoire restriction of infiltrating T cells.^35^

Furthermore, we were able to identify TCRVβ/Vα pairs in FFPE needle biopsies from patients with breast cancer. FFPE samples often show lower RNA transcript quality and give lower yields compared to fresh frozen samples.^25^^,^^36^^,^^37^ Importantly, the ability to map TCRVβ/Vα pairs in FFPE sections allows for the use of archival biobank samples.

One limitation of targeted approaches is the restriction on available targets due to sequences’ properties (e.g. length, GC content, homology, etc). In this study, we designed a probe panel covering alpha, beta and delta constant TCR genes, as well as the majority of alpha, beta, gamma and delta variable genes. Due to the short length and high homology of Joining and Diversity genes, probe design was only possible for a subset of alpha and beta Joining genes. Consequently, our approach does not capture full TCR clonotype information, as defined by CDR3 sequences, but instead relies on variable gene pairing (TCRVβ/Vα pairs) as a proxy. Although probe design for known CDR3 regions is theoretically possible, it requires prior knowledge from sequencing data and sequence compatibility with probe design requirements. By successfully detecting both alpha and beta variable genes at single-cell level, we were able to identify TCRVβ/Vα pairs and investigated expansions of those cells. Since cells with identical CDR3s would also share the same TCRVβ/Vα combinations, a TCRVβ/Vα expansion would indicate, but not explicitly define, potential CDR3 sharing. Future studies integrating spatial approaches with sequencing-based spatial methods or other comprehensive repertoire profiling methods will be essential to determine how well variable gene pairing reflects CDR3 clonotypic diversity and to further extend the biological interpretation of these findings.

It is important to emphasise that all TCR targeting methodologies have their own advantages and limitations, and should therefore be viewed as complementary rather than interchangeable. While targeted and imaging-based approaches necessarily introduce blind spots due to probe design constraints, they enable direct mapping of clonotype-associated gene expression to tissue architecture, and thereby allow the study of spatial TCRVβ/Vα expansion at single-cell resolution. In contrast, bulk repertoire sequencing methods (e.g. Adaptive ImmunoSeq) provide extensive coverage of TCR diversity, but lack spatial context and single-cell localisation. Sequencing-based spatial methods enable transcriptome-wide profiling, and recently, near single-cell resolution,^12^^,^^38^ while (depending on the platform) also allow identification of TCR sequences, including CDR3 regions. However, these approaches may require computational pairing of alpha and beta chains^11^^,^^15^^,^^16^ or may still capture transcripts from adjacent cells.^12^^,^^38^ Together, these considerations highlight that no single platform is sufficient to fully resolve TCR diversity and spatial organisation, and that integrating multiple approaches provides a more comprehensive view than any individual method alone.

The current study lacks direct benchmarking against alternative spatial transcriptomics platforms or matched single-cell RNA sequencing datasets. Such comparisons would enable a quantitative assessment of sensitivity, specificity, and detection efficiency across technologies. However, these analyses would require matched samples and dedicated experimental design; therefore future studies using multi-modal approaches and prospectively collected samples will be essential to systematically evaluate performance across platforms and further refine the interpretation of spatial TCR profiling.

Our proof-of-concept study made use of a limited number of samples. We used two NSCLC surgical specimens of known clonality and histopathology and a small FFPE cohort of clinical needle biopsies from patients with breast cancer. We were able to confirm known biological patterns and demonstrate the types of analysis one can do to explore TCR diversity in such samples. We successfully demonstrate that paired TCRVα/Vβ gene usage at single-cell level can be robustly detected, spatially resolved, and integrated with cell-type annotations, providing meaningful insights into local TCR diversity. Our method can be applied in fresh frozen and FFPE samples, making the use of archival clinical samples possible. While our descriptive observations are in line with previously reported biological patterns, drawing definitive biological conclusions will require validation in larger cohorts.

Our approach establishes a foundation for future studies and, given its flexibility in probe panel design, enables the co-characterisation of the tumour immune microenvironment with additional targets for specific immune cell types and functional states. Extensive characterisation of all immune cell types and functional states was beyond the scope of the present study, however, such analyses will be required to support broader biological interpretations and would benefit from expanded and/or customisable probe panels and larger patient cohorts, representing important directions for future research.

## Contributors

C.S., M.N. and A.M. conceived the study. A.M., M.C., and C.S. developed the methodology of the study. A.M. and M.C. performed experiments. A.M., M.C., A. Met., T.H. and H.Y. developed software. M.N., C.S., P.M., I.Z., K.W. and T.F. contributed reagents and materials. T.H., M.L., I.Z., K.W., T.F. and P.M. contributed with expertise on cancer histology and pathology. K.L. contributed with immunological expertise. M.N. acquired funding. A.M. prepared the figures and wrote the paper. A.M., M.N., and C.S. critically revised the article for important intellectual content. The corresponding authors A.M., M.N. and C.S. had full access to all the data in the study, verified the underlying data and take responsibility for the integrity of the data and the accuracy of the data analysis. All authors read and approved the final version of the manuscript.

## Data sharing statement

The data from the NSCLC biopsies can be visualised and explored through the TissUUmaps web-based tool, accessible at https://re0978c72.serve.scilifelab.se. Similarly, the data from the PREDIX LumB biopsies are accessible at https://r0928505c.serve.scilifelab.se.

The raw data from Xenium experiments (several terabytes) are available from the corresponding author upon reasonable request.

## Declaration of interests

Mats Nilsson has been a scientific advisor for 10X genomics.

Ioannis Zerdes has received institutional research grants from Gilead Sciences and honoraria paid to his institution from Novartis. He has received personal Honoraria from BioMed Central, part of Springer Nature Group.

The rest of the authors declare no competing interests.

## Appendix A Supplementary data

Fig. S1 **Principal workflow of Xenium.** The Xenium workflow begins with sample preparation, where fresh frozen (FF) or formalin-fixed paraffin-embedded (FFPE) tissue samples are mounted on Xenium slides. FF samples are fixed and permeabilized, while FFPE samples are deparaffinized and decrosslinked. Following this, probe hybridisation, ligation, and rolling circle amplification (RCA) are performed. Imaging is then conducted in cycles on the Xenium Analyser, where fluorescently labelled probes for RNA target sequences are cycled in, imaged, and removed. Data are captured across multiple Z-planes with a 0.75 μm step size and fluorescence channels, producing a spatial map of transcripts in the tissue section (the figure is courtesy of 10X Genomics).

Fig. S2 **Cell type distribution in NSCLC.** Clustering results for two NSCLC biopsies. A) UMAP representation of identified cell types in adenocarcinoma and squamous cell lung carcinoma. Abbreviations: CAFs: Cancer associated fibroblasts, CD4 TLS T cells: CD4 tertiary lymphoid structure associated cells, CD4 act T cells: CD4 activated T cells, M2 MΦ: M2 macrophages, MDCs: myeloid dendritic cells, Squamous CC: Squamous cell carcinoma. B) UMAP illustration of cell distribution per sample. C) Cell distribution of identified cell types in the NSCLC samples. Benign alveolar cells: Benign alveolar cells 1–2, Cancer AC: Cancer 1–10, CD8 T cells: CD8 T cells 1–4, Endothelial cells: Endothelial cells 1–2, Myofibroblasts: Myofibroblasts 1–2, Myeloid cells: Macrophages, M2 MΦ, Myeloid cells, CD4 TLS T cells: CD4 TLS T cells 1–2, Plasma/ B cells: Plasma/ B cells 1–2, Squamous CC: Squamous CC 1–5.

Fig. S3 **Density and diversity plots for TCR variable genes in Squamous cell lung carcinoma.** Density plot for A) Beta variable and B) Alpha variable TCR genes. Diversity plot for C) TRBV and D) TRAV genes, estimated as the percentage of expression of the dominant variable gene over the rest variable genes in one bin. Only bins where the sum of TRBV or TRAV counts is >10 are considered. Scale bar = 1000 μm.

Fig. S4 **Density and diversity plots for TCR variable genes in adenocarcinoma.** Density plot for A) Beta variable and B) Alpha variable TCR genes. Diversity plot for C) TRBV and D) TRAV genes, estimated as the percentage of expression of the dominant variable gene over the rest variable genes in one bin. Only bins where the sum of TRBV or TRAV counts is >10 are considered. Scale bar = 1000 μm.

Fig. S5 **TCRVβ/Vα pair identification in adenocarcinoma and squamous cell lung carcinoma.** A) Percentage of identified TCRVβ/Vα pairs against all cells per cell type. B) Number of cells identified with TCR chain gene combinations. ab multi chain: two Vα and two Vβ genes identified, ab single pair: one Vα and one Vβ gene (TCRVβ/Vα pair), extra alpha: two Vα genes and one Vβ gene, extra beta: one Vα gene and two Vβ genes, ab unknown: the remaining cases. Similarly, for γδ T cells, extra delta: one Vγ and two Vδ genes, extra gamma: two Vγ and one Vδ gene, gd multi chain: two Vγ and two Vδ genes, gd single pair: one Vγ and one Vδ genes, gd unknown: the remaining cases. Only TCRVβ/Vα pairs were used for further analysis in this study.

Fig. S6 **T cell populations and TCRVβ/Vα pair distribution in squamous cell lung carcinoma.** A) Neighbourhood analysis of the identified T cell subtypes and their most abundant TCRVβ/Vα pairs in relation with the rest cell types on the tissue within a 5-cell radius. B) Number of cells in identified T cell subtypes. C) Number of TCRVβ/Vα pairs cells per T cell subtype. D) Proportions of identified TCRVβ/Vα pairs in T cell subsets. The three most abundant TCRVβ/Vα pairs of each T cell subtype are colour-coded and the rest of the TCRVβ/Vα pairs represented as Others.

Fig. S7 **Identified spatial domains in SqCC NSCLC biopsy.** CD8 T cells: CD8 T cells 1–4, Squamous CC: Squamous CC 1–5, Cancer AC: Cancer 1–10, Myofibroblasts: Myofibroblasts 1–2, Plasma/ B cells: Plasma/ B cells 1–2, CD4 act T cells: CD4 act T cells, Myeloid cells: Macrophages, Myeloid cells, M2_MΦ, Benign alveolar cells: Benign alveolar cells 1–2, MDCs: MDCs, CD4 TLS T cells: CD4 TLS T cells 1–2, Endothelial cells: Endothelial cells 1–2, SERPINB9 endothelial cells, CAFs: CAFs.
