# Supplementary material for: Spatially resolved T cell receptor diversity mapping uncovers variability of the cancer immune microenvironment
Source: eBioMedicine. 2026 Apr 24;127:106264. doi: 10.1016/j.ebiom.2026.106264 (PMC13127328; doi:10.1016/j.ebiom.2026.106264)
Supplement: Fig. S1 — Principal workflow of Xenium. The Xenium workflow begins with sample preparation, where fresh frozen (FF) or formalin-fixed paraffin-embedded (FFPE) tissue samples are mounted on Xenium slides. FF samples are fixed and permeabilized, while FFPE samples are deparaffinized and decrosslinked. Following this, probe hybridisation, ligation, and rolling circle amplification (RCA) are performed. Imaging is then conducted in cycles on the Xenium Analyser, where fluorescently labelled probes for RNA target sequences are cycled in, imaged, and removed. Data are captured across multiple Z-planes with a 0.75 μm step size and fluorescence channels, producing a spatial map of transcripts in the tissue section (the figure is courtesy of 10X Genomics). [file mmc1.pdf]

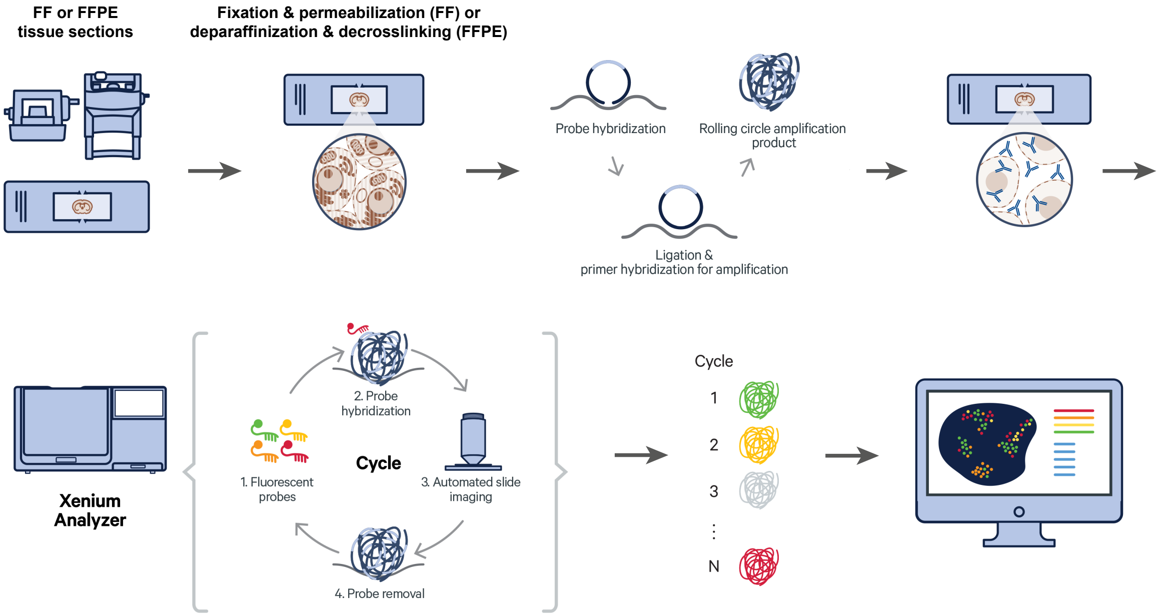
FF or FFPE
tissue sections
Fixation & permeabilization (FF) or
deparaffinization & decrosslinking (FFPE)
Probe hybridization
Rolling circle amplification
product
Ligation &
primer hybridization for amplification
Xenium
Analyzer
2. Probe
hybridization
1. Fluorescent
probes
Cycle
3. Automated slide
imaging
4. Probe removal
Cycle
1
2
3
⋮
N
