# Supplementary figures and images for: Spatially resolved T cell receptor diversity mapping uncovers variability of the cancer immune microenvironment
Source: eBioMedicine. 2026 Apr 24;127:106264. doi: 10.1016/j.ebiom.2026.106264 (PMC13127328; doi:10.1016/j.ebiom.2026.106264)

A

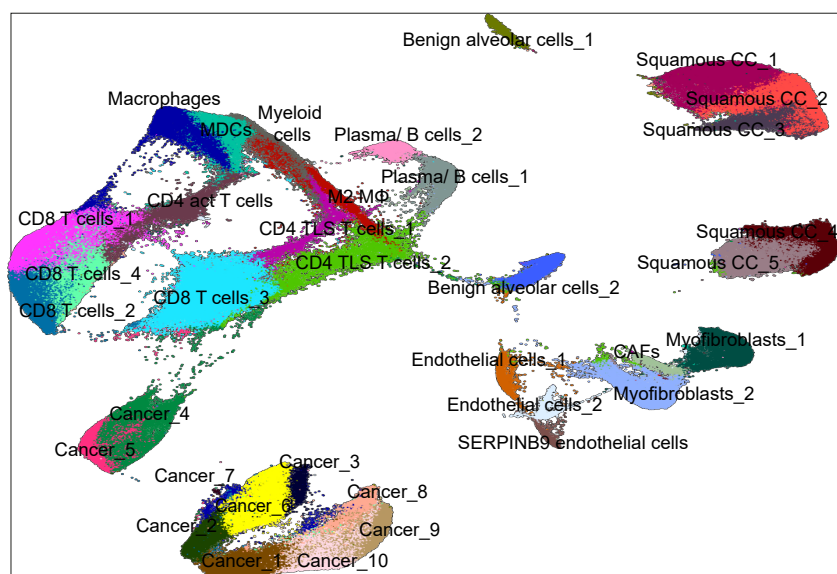

B

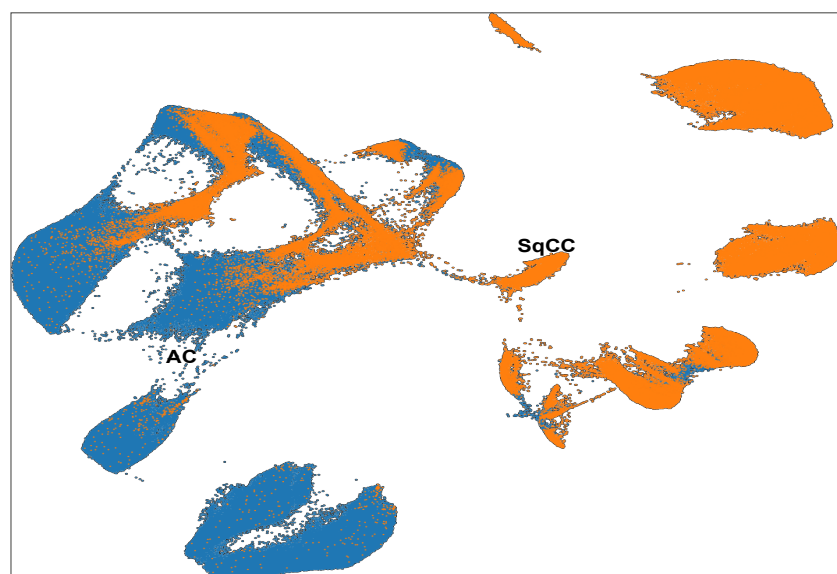

C

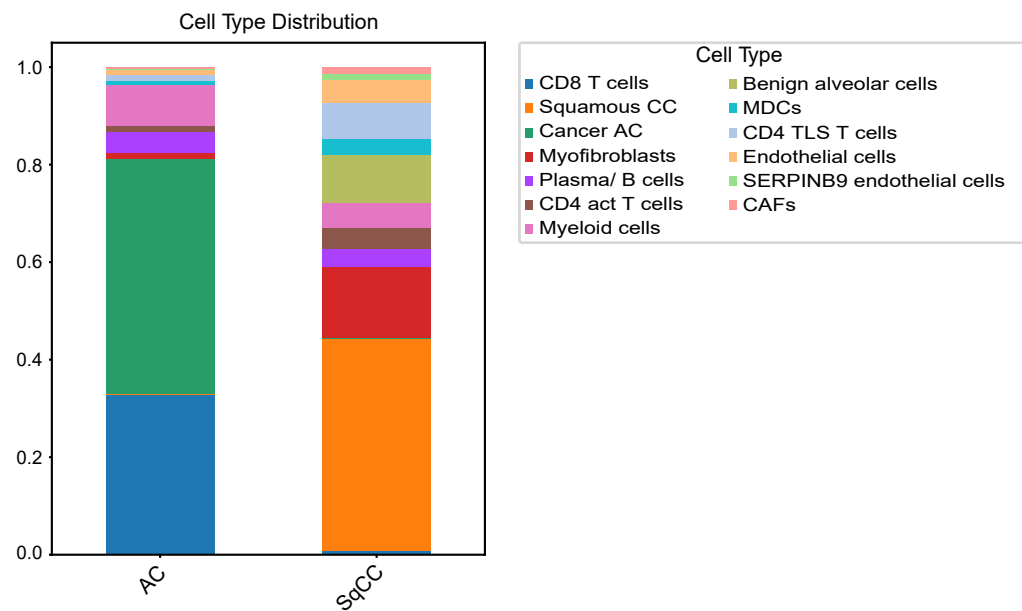

Supplement: Fig. S2 — Cell type distribution in NSCLC. Clustering results for two NSCLC biopsies. A) UMAP representation of identified cell types in adenocarcinoma and squamous cell lung carcinoma. Abbreviations: CAFs: Cancer associated fibroblasts, CD4 TLS T cells: CD4 tertiary lymphoid structure associated cells, CD4 act T cells: CD4 activated T cells, M2 MΦ: M2 macrophages, MDCs: myeloid dendritic cells, Squamous CC: Squamous cell carcinoma. B) UMAP illustration of cell distribution per sample. C) Cell distribution of identified cell types in the NSCLC samples. Benign alveolar cells: Benign alveolar cells 1–2, Cancer AC: Cancer 1–10, CD8 T cells: CD8 T cells 1–4, Endothelial cells: Endothelial cells 1–2, Myofibroblasts: Myofibroblasts 1–2, Myeloid cells: Macrophages, M2 MΦ, Myeloid cells, CD4 TLS T cells: CD4 TLS T cells 1–2, Plasma/ B cells: Plasma/ B cells 1–2, Squamous CC: Squamous CC 1–5. [file mmc2.pdf]

**A**

Density plot TRBVs &gt;10

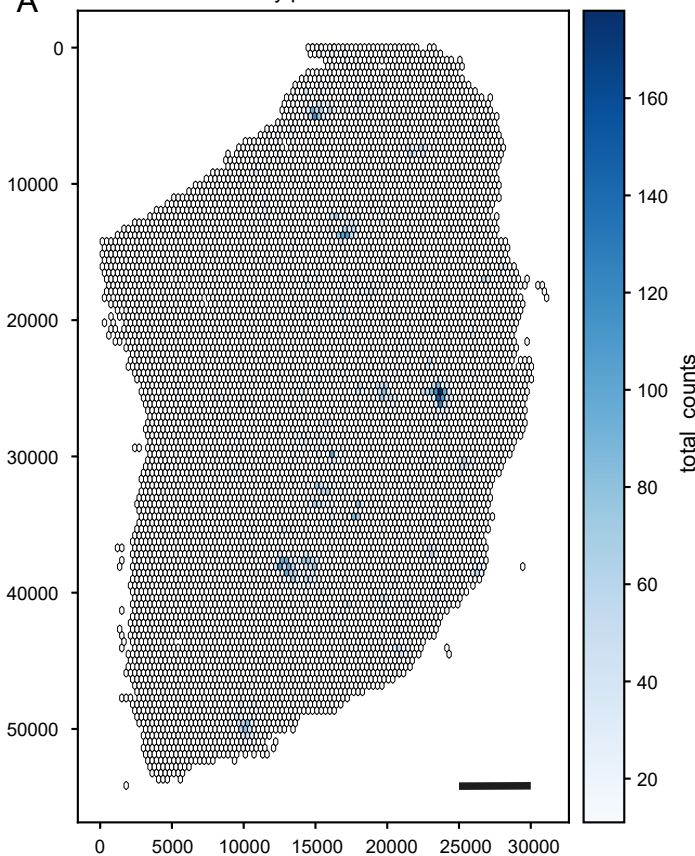**B**

Density plot TRAVs &gt;10

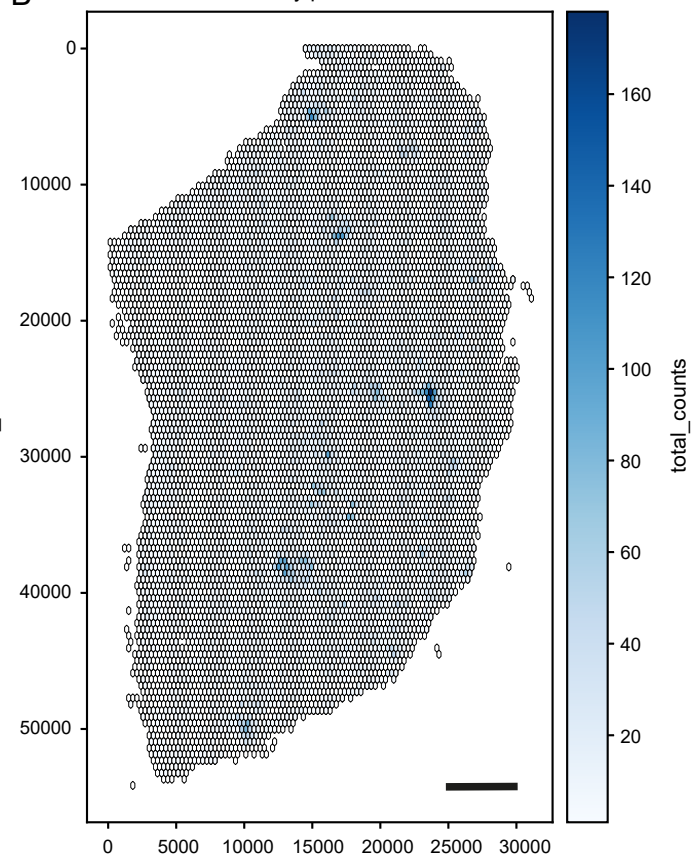**C**

Max fraction TRBVs &gt;10

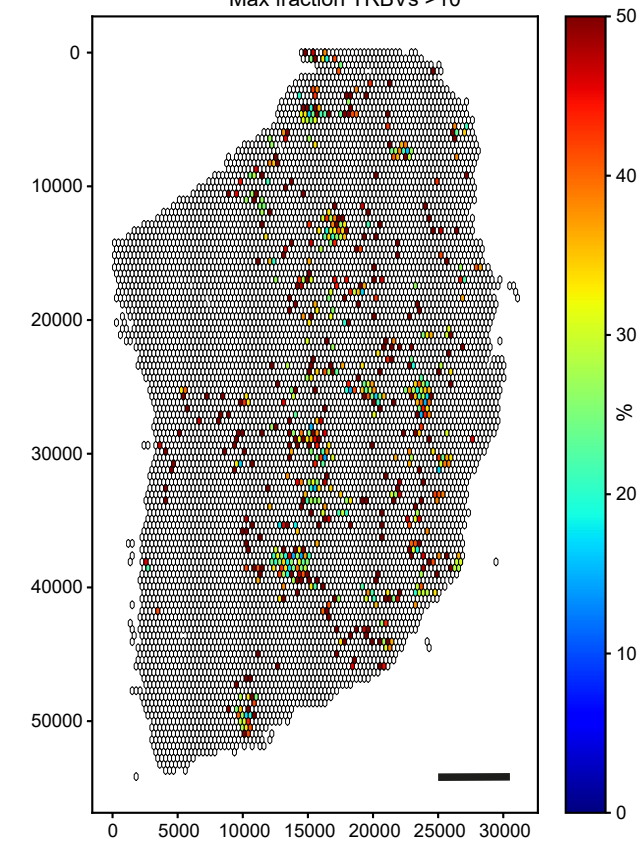**D**

Max fraction TRAVs &gt;10

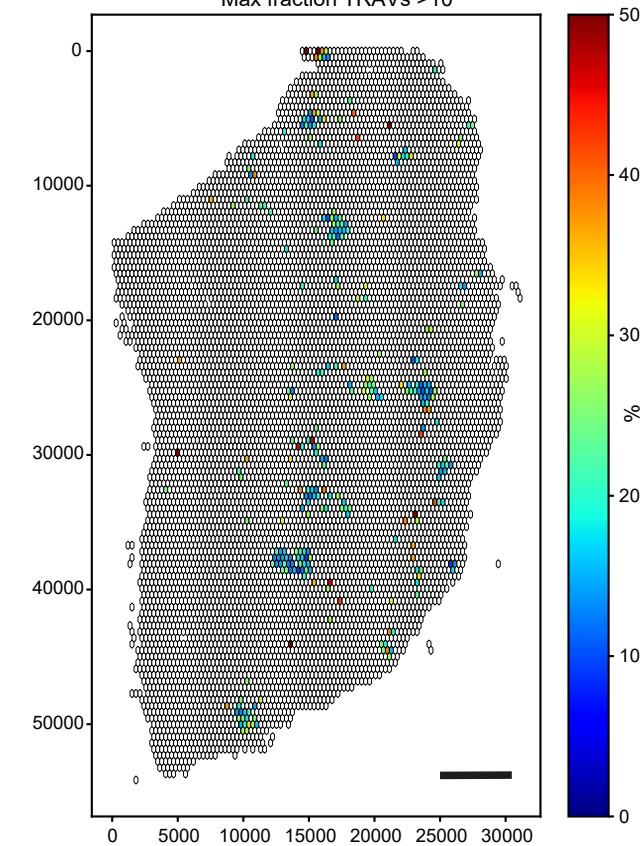

Supplement: Fig. S3 — Density and diversity plots for TCR variable genes in Squamous cell lung carcinoma. Density plot for A) Beta variable and B) Alpha variable TCR genes. Diversity plot for C) TRBV and D) TRAV genes, estimated as the percentage of expression of the dominant variable gene over the rest variable genes in one bin. Only bins where the sum of TRBV or TRAV counts is >10 are considered. Scale bar = 1000 μm. [file mmc3.pdf]

A

Density plot TRBVs &gt;10

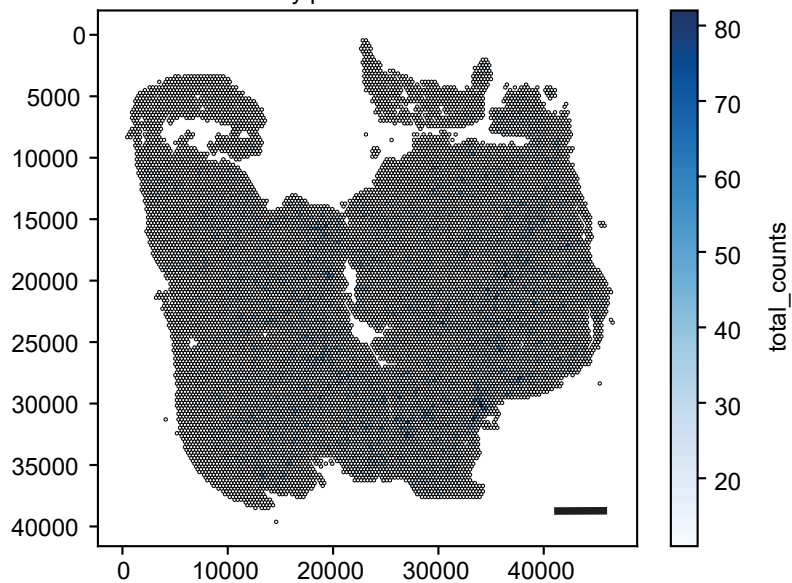

B

Density plot TRAVs &gt;10

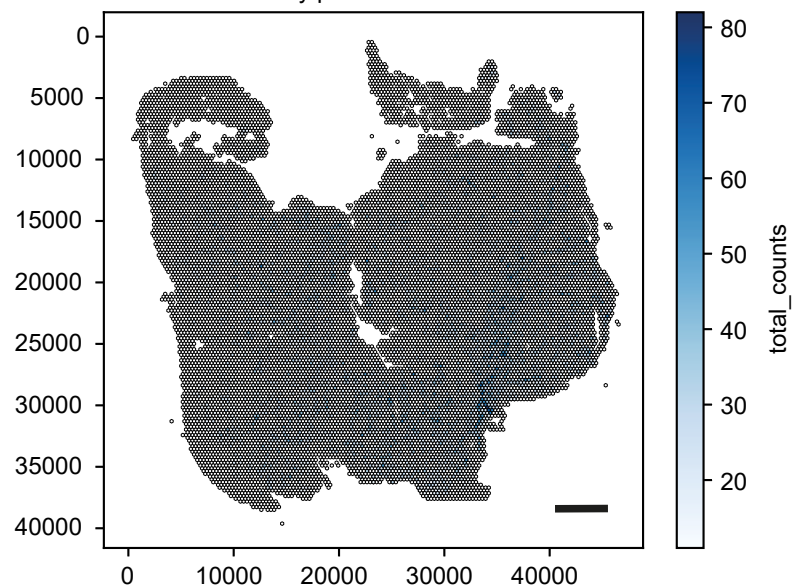

C

Max fraction TRBVs &gt;10

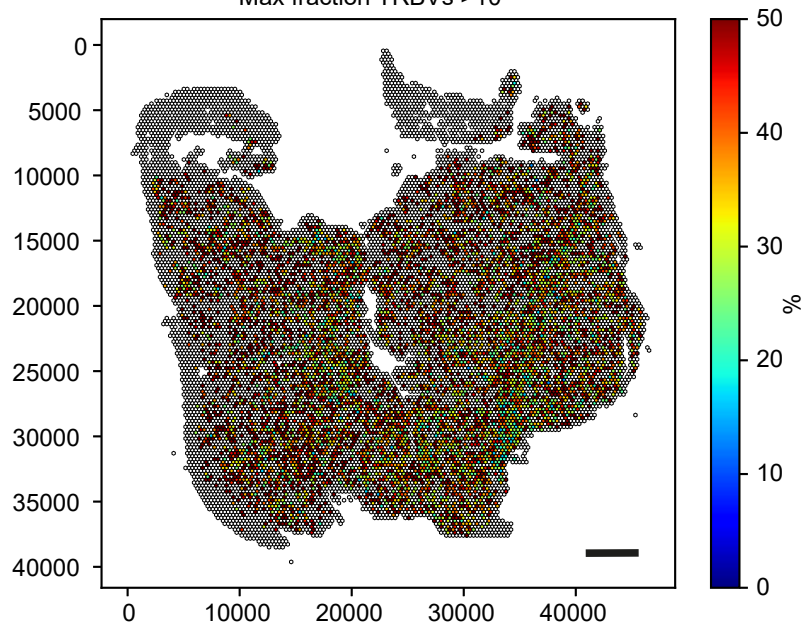

D

Max fraction TRAVs &gt;10

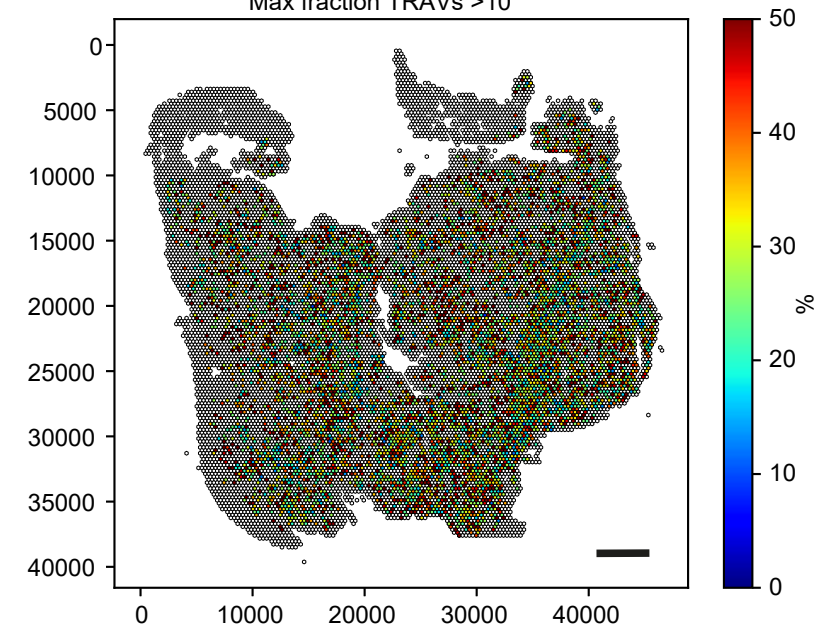

Supplement: Fig. S4 — Density and diversity plots for TCR variable genes in adenocarcinoma. Density plot for A) Beta variable and B) Alpha variable TCR genes. Diversity plot for C) TRBV and D) TRAV genes, estimated as the percentage of expression of the dominant variable gene over the rest variable genes in one bin. Only bins where the sum of TRBV or TRAV counts is >10 are considered. Scale bar = 1000 μm. [file mmc4.pdf]

A

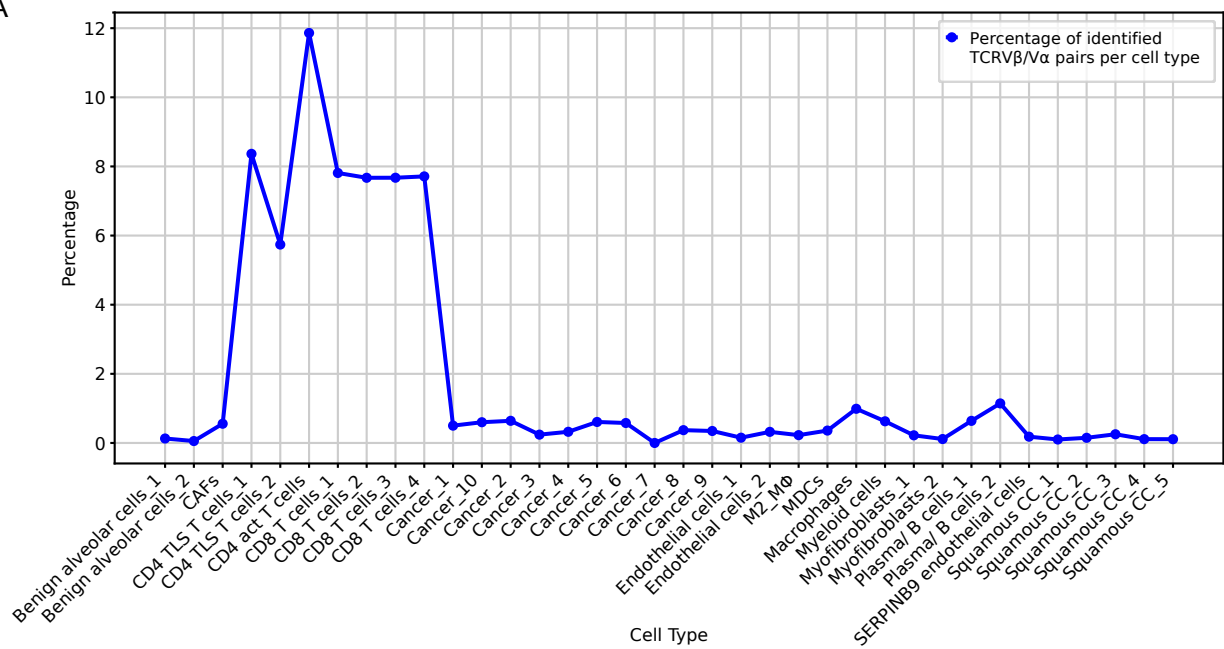

B

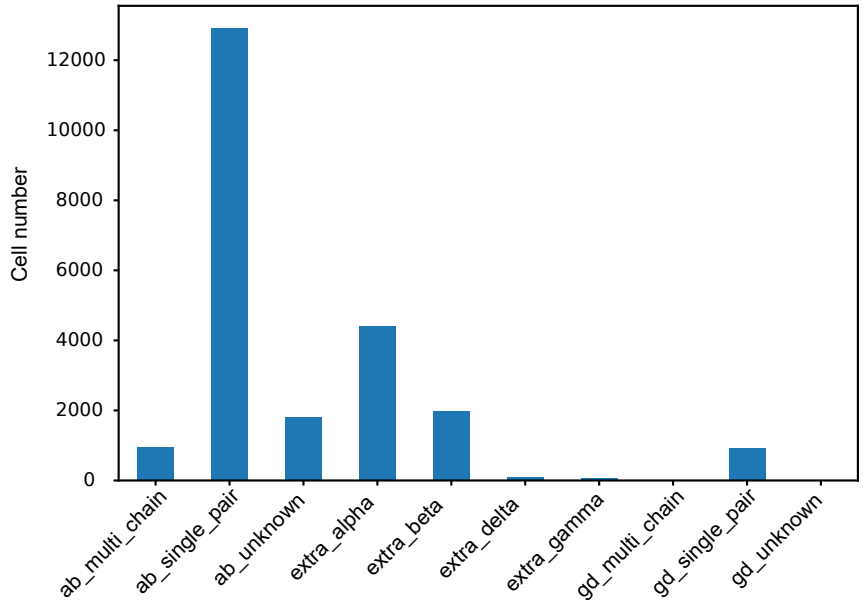

Supplement: Fig. S5 — TCRVβ/Vα pair identification in adenocarcinoma and squamous cell lung carcinoma. A) Percentage of identified TCRVβ/Vα pairs against all cells per cell type. B) Number of cells identified with TCR chain gene combinations. ab multi chain: two Vα and two Vβ genes identified, ab single pair: one Vα and one Vβ gene (TCRVβ/Vα pair), extra alpha: two Vα genes and one Vβ gene, extra beta: one Vα gene and two Vβ genes, ab unknown: the remaining cases. Similarly, for γδ T cells, extra delta: one Vγ and two Vδ genes, extra gamma: two Vγ and one Vδ gene, gd multi chain: two Vγ and two Vδ genes, gd single pair: one Vγ and one Vδ genes, gd unknown: the remaining cases. Only TCRVβ/Vα pairs were used for further analysis in this study. [file mmc5.pdf]

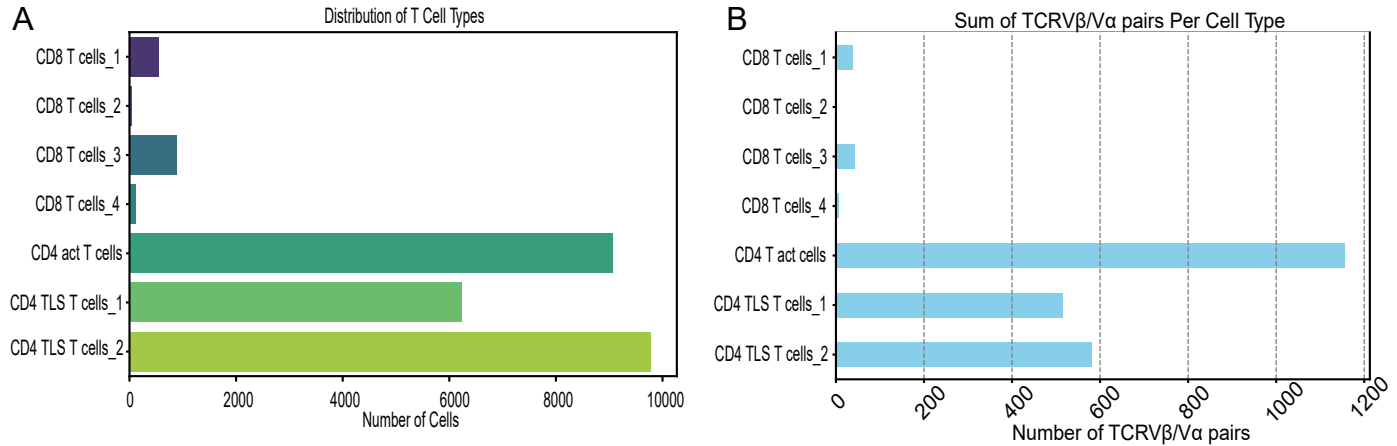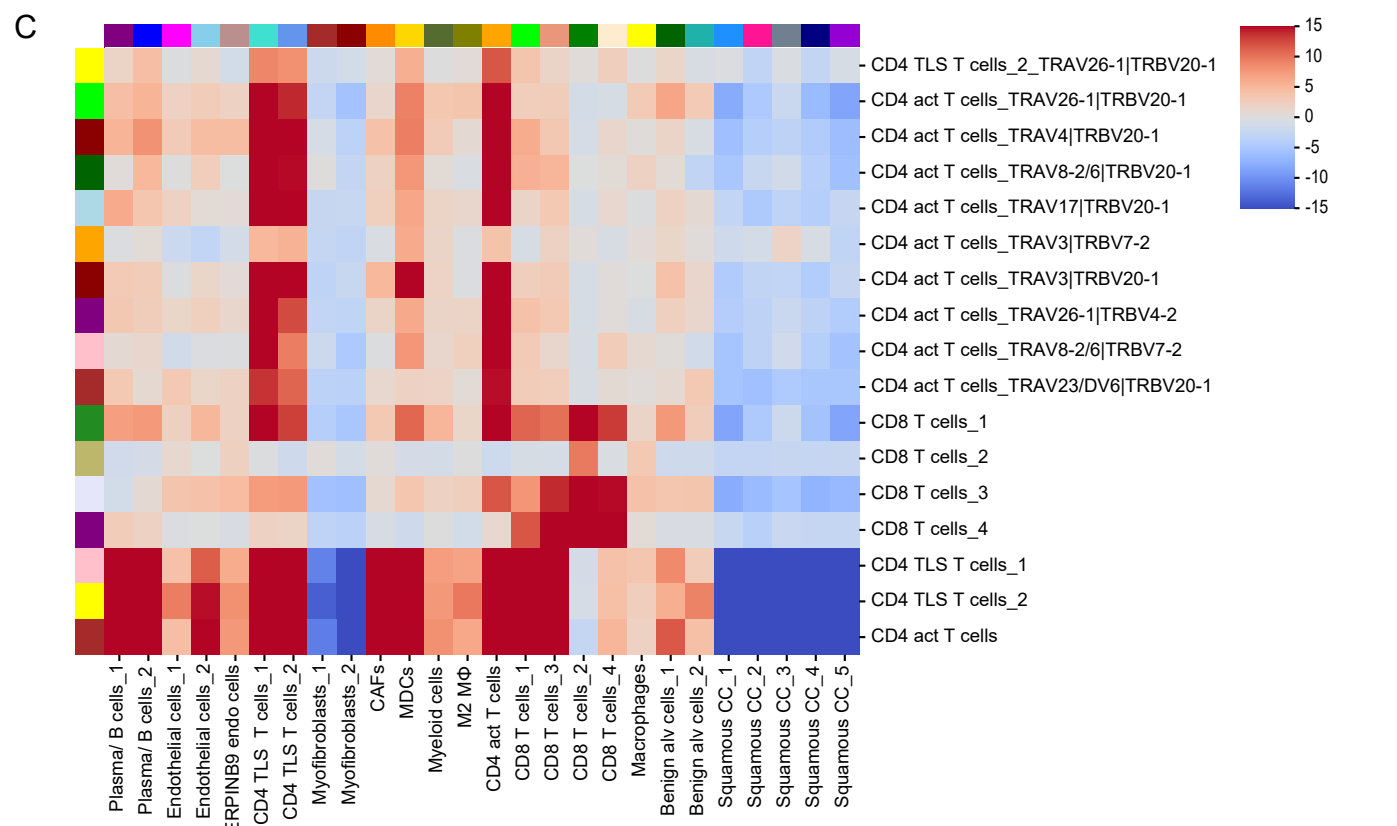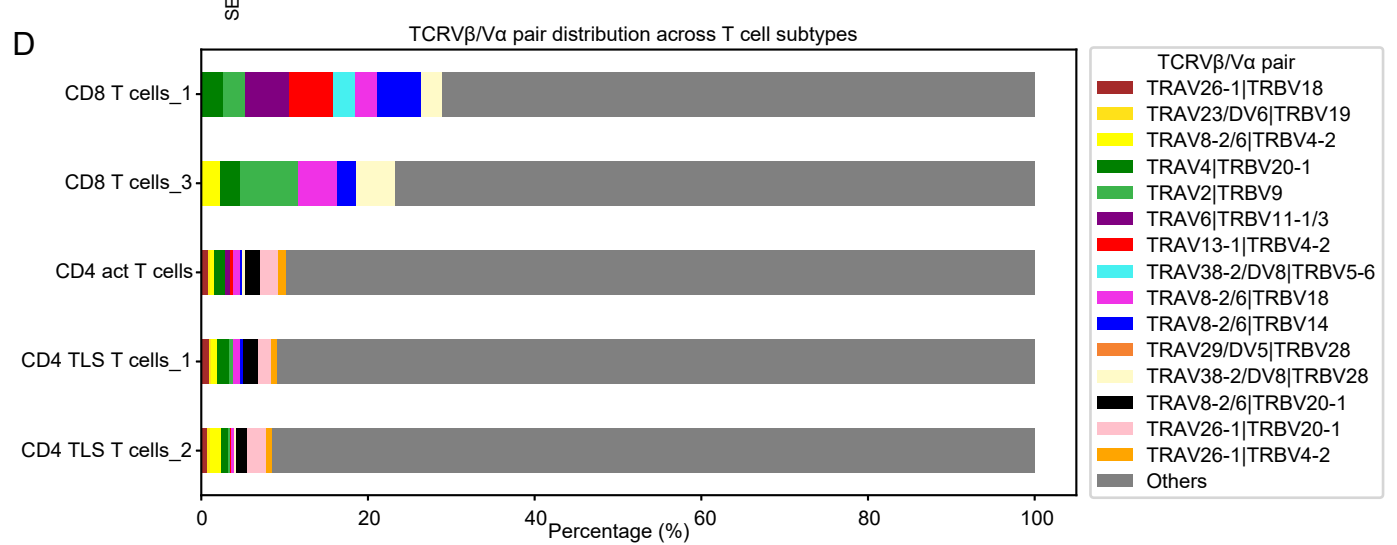

Supplement: Fig. S6 — T cell populations and TCRVβ/Vα pair distribution in squamous cell lung carcinoma. A) Neighbourhood analysis of the identified T cell subtypes and their most abundant TCRVβ/Vα pairs in relation with the rest cell types on the tissue within a 5-cell radius. B) Number of cells in identified T cell subtypes. C) Number of TCRVβ/Vα pairs cells per T cell subtype. D) Proportions of identified TCRVβ/Vα pairs in T cell subsets. The three most abundant TCRVβ/Vα pairs of each T cell subtype are colour-coded and the rest of the TCRVβ/Vα pairs represented as Others. [file mmc6.pdf]

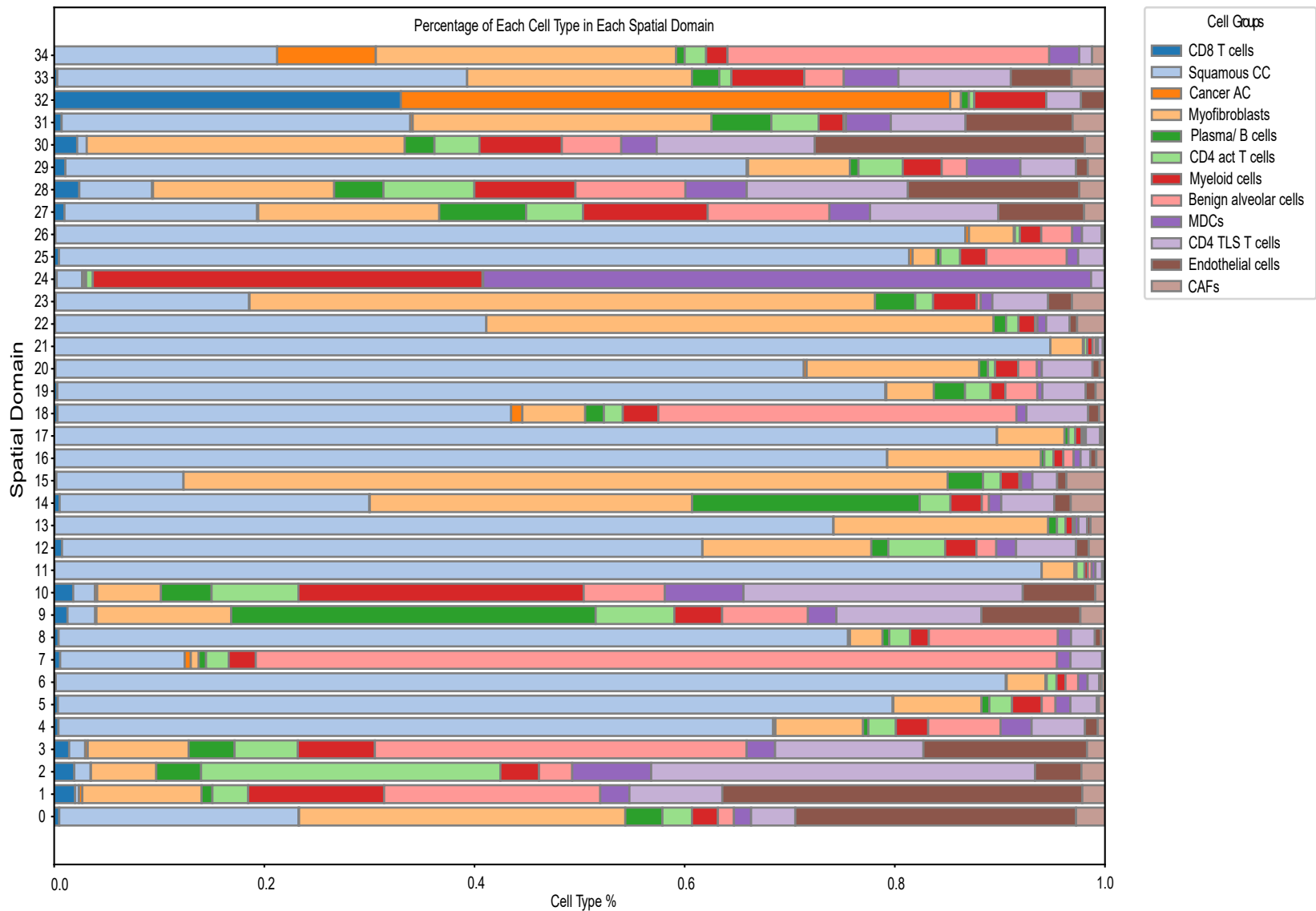

Supplement: Fig. S7 — Identified spatial domains in SqCC NSCLC biopsy. CD8 T cells: CD8 T cells 1–4, Squamous CC: Squamous CC 1–5, Cancer AC: Cancer 1–10, Myofibroblasts: Myofibroblasts 1–2, Plasma/ B cells: Plasma/ B cells 1–2, CD4 act T cells: CD4 act T cells, Myeloid cells: Macrophages, Myeloid cells, M2_MΦ, Benign alveolar cells: Benign alveolar cells 1–2, MDCs: MDCs, CD4 TLS T cells: CD4 TLS T cells 1–2, Endothelial cells: Endothelial cells 1–2, SERPINB9 endothelial cells, CAFs: CAFs. [file mmc7.pdf]
