# Supplementary material for: Spatially resolved T cell receptor diversity mapping uncovers variability of the cancer immune microenvironment
Source: eBioMedicine. 2026 Apr 24;127:106264. doi: 10.1016/j.ebiom.2026.106264 (PMC13127328; doi:10.1016/j.ebiom.2026.106264)
Supplement: Supplementary Tables [file mmc8.docx]

| **Supplementary Table 1:** Demographic data of the patients with Non-small cell lung cancer (NSCLC). Patients were operated at the Uppsala University Hospital in Sweden, between 2006 and 2010. | | |
| --- | --- | --- |
| Histology | Adenocarcinoma | Squamous cell lung carcinoma |
| Age | 74 | 69 |
| Sex | Female | Female |
| Smoking status | Former smoker | Never smoked |
| Stage (8^th^ edition) | 2A | 1A3 |
| Performance status (WHO) | 0 | 1 |

**Supplementary Table 2:** Demographic data of the patients with breast cancer included in PREDIX Lum B clinical trial.

| **Treatment** | Palbociclib in combination with endocrine therapy |
| --- | --- |
| **Median age** | 53,5 |
| < 50 | 2 |
| ≥ 50 | 4 |
| **Tumour size** | |
| <2 cm | 2 |
| 2-5 cm | 2 |
| >5 cm | 2 |
| **Sex** | |
| Female | 6 |
| Male | 0 |
| **Node status** | |
| Positive | 5 |
| Negative | 1 |
| **ER status** |  |
| Positive | 6 |
| Negative | 0 |
| **HER2 status** |  |
| Positive | 0 |
| Negative | 6 |

**Supplementary Table 3:** Probe coverage for alpha, beta, gamma and delta chain variable and constant T cell receptor genes.

| **Alpha chain** | **Available probe** | **Beta chain** | **Available probe** | **Gamma chain** | **Available probe** | **Delta chain** | **Available probe** |
| --- | --- | --- | --- | --- | --- | --- | --- |
| **Variable** |  | **Variable** |  | **Variable** |  | **Variable** |  |
| TRAV1-1 | + | TRBV2 | + | TRGV2 | +  Common  probe | TRDV1 | + |
| TRAV1-2 | + | TRBV3-1 | + | TRGV4 |  | TRDV2 | + |
| TRAV2 | + | TRBV4-2 | + | TRGV4 | +  Common  probe | TRDV3 | + |
| TRAV3 | + | TRBV5-1 | + | TRGV5 |  |  |  |
| TRAV4 | + | TRBV5-4 | - | TRGV8 | + | **Constant** |  |
| TRAV5 | + | TRBV5-5 | - | TRGV9 | + | TRDC | + |
| TRAV6 | + | TRBV5-6 | - |  |  |  |  |
| TRAV7 | + | TRBV5-8 | + | **Constant** |  |  |  |
| TRAV8-1 | + | TRBV6-1 | - | TRGC1 | - |  |  |
| TRAV8-3 | - | TRBV6-2 | - | TRGC2 | - |  |  |
| TRAV8-2 | +  Common  probe | TRBV6-3 | - |  |  |  |  |
| TRAV8-4 |  | TRBV6-4 | + |  |  |  |  |
| TRAV8-6 |  | TRBV6-5 | - |  |  |  |  |
| TRAV9-1 | + | TRBV6-6 | - |  |  |  |  |
| TRAV9-2 | + | TRBV6-8 | + |  |  |  |  |
| TRAV10 | + | TRBV6-9 | - |  |  |  |  |
| TRAV12-1 | + | TRBV7-2 | + |  |  |  |  |
| TRAV12-2 | + | TRBV7-3 | + |  |  |  |  |
| TRAV12-3 | + | TRBV7-4 | - |  |  |  |  |
| TRAV13-1 | + | TRBV7-6 | - |  |  |  |  |
| TRAV13-2 | + | TRBV7-7 | - |  |  |  |  |
| TRAV14/DV4 | + | TRBV7-8 | - |  |  |  |  |
| TRAV16 | + | TRBV7-9 | + |  |  |  |  |
| TRAV17 | - | TRBV9 | + |  |  |  |  |
| TRAV18 | - | TRBV10-1 | - |  |  |  |  |
| TRAV19 | + | TRBV10-2 | - |  |  |  |  |
| TRAV20 | + | TRBV10-3 | + |  |  |  |  |
| TRAV21 | + | TRBV11-1 | +  Common  probe |  |  |  |  |
| TRAV22 | + | TRBV11-2 |  |  |  |  |  |
| TRAV23/DV6 | + | TRBV11-3 |  |  |  |  |  |
| TRAV24 | + | TRBV12-3 | +  Common  probe |  |  |  |  |
| TRAV25 | + | TRBV12-4 |  |  |  |  |  |
| TRAV26-1 | + | TRBV12-5 | + |  |  |  |  |
| TRAV26-2 | + | TRBV13 | + |  |  |  |  |
| TRAV27 | + | TRBV14 | + |  |  |  |  |
| TRAV29/DV5 | + | TRBV15 | + |  |  |  |  |
| TRAV30 | + | TRBV16 | + |  |  |  |  |
| TRAV34 | + | TRBV18 | + |  |  |  |  |
| TRAV35 | + | TRBV19 | + |  |  |  |  |
| **Alpha chain** | **Available probe** | **Beta chain** | **Available probe** | **Gamma chain** | **Available probe** | **Delta chain** | **Available probe** |
| **Variable** |  | **Variable** |  | **Variable** |  | **Variable** |  |
| TRAV36/DV7 | + | TRBV20-1 | - |  |  |  |  |
| TRAV38-1 | - | TRBV24-1 | + |  |  |  |  |
| TRAV38-2/DV8 | + | TRBV25-1 | + |  |  |  |  |
| TRAV39 | + | TRBV27 | + |  |  |  |  |
| TRAV40 | + | TRBV28 | + |  |  |  |  |
| TRAV41 | + | TRBV29-1 | - |  |  |  |  |
|  |  | TRBV30 | + |  |  |  |  |
| **Constant** |  |  |  |  |  |  |  |
| TRAC | + | **Constant** |  |  |  |  |  |
|  |  | TRBC1 | +  Common  probe |  |  |  |  |
|  |  | TRBC2 |  |  |  |  |  |

| **Supplementary Table 4:** Target sequences for TCR genes. | | |
| --- | --- | --- |
|  | TCR Gene name | Target sequence 5’->3’ |
| 1 | TRAC_00 | TCCAGAACCCTGACCCTGCCGTGTACCAGCTGAGAGACTC |
| 2 | TRAJ18_00 | CTATACTTTGGAAGAGGAACTCAGTTGACTGTCTGGCCTG |
| 3 | TRAJ20_00 | GCTCAGCTTTGGAGCCGGAACCACAGTAACTGTAAGAGCA |
| 4 | TRAJ31_00 | CTCATGTTTGGAGATGGAACTCAGCTGGTGGTGAAGCCCA |
| 5 | TRAJ32_00 | CTCATCTTTGGAACTGGCACTCTGCTTGCTGTCCAGCCAA |
| 6 | TRAJ39_00 | GAATAATAATGCAGGCAACATGCTCACCTTTGGAGGGGGA |
| 7 | TRAJ45_00 | TGTATTCAGGAGGAGGTGCTGACGGACTCACCTTTGGCAA |
| 8 | TRAJ47_00 | GGTCTTTGGCGCAGGAACCATTCTGAGAGTCAAGTCCTAT |
| 9 | TRAJ54_00 | GCTGGTATTTGGCCAAGGAACCAGGCTGACTATCAACCCA |
| 10 | TRAV1-1_00 | TCATTCCTTAGTCGCTCTGATAGTTATGGTTACCTCCTTC |
| 11 | TRAV1-2_00 | CCTTAGTCGGTCTAAAGGGTACAGTTACCTCCTTTTGAAG |
| 12 | TRAV1-2_01 | AAACATTGACCAGCCCACTGAGATGACAGCTACGGAAGGT |
| 13 | TRAV10_00 | CAGTGAGAACACAAAGTCGAACGGAAGATATACAGCAACT |
| 14 | TRAV10_01 | TGGATGCAGACACAAAGCAAAGCTCTCTGCACATCACAGC |
| 15 | TRAV10_02 | GGAGAGGTCCTGTTTCCCTGACAATCATGACTTTCAGTGA |
| 16 | TRAV12-1_00 | GGATTGCAGGAAAGAACCTAAGTTGCTGATGTCCGTATAC |
| 17 | TRAV12-2_00 | TGCCTCTCTCAACTGCACTTACAGTGACCGAGGTTCCCAG |
| 18 | TRAV12-3_00 | CAATACTTCATGTGGTACAGACAGTATTCCAGAAAAGGCC |
| 19 | TRAV13-1_00 | AATTGCTGTTACATTGAACAAGACAGCCAAACATTTCTCC |
| 20 | TRAV13-2_00 | TTGAATAAGACAGTGAAACATCTCTCTCTGCAAATTGCAG |
| 21 | TRAV14/DV4_00 | CCCAGAAGATAACTCAAACCCAACCAGGAATGTTCGTGCA |
| 22 | TRAV14/DV4_01 | TGCAGGAAAAGGAGGCTGTGACTCTGGACTGCACATATGA |
| 23 | TRAV14/DV4_02 | TCCGCCAACCTTGTCATCTCCGCTTCACAACTGGGGGACT |
| 24 | TRAV16_00 | TATGTCCAGTACTCCAGACAACGCCTCCAGTTACTCTTGA |
| 25 | TRAV16_01 | TGAGACACATCTCTAGAGAGAGCATCAAAGGCTTCACTGC |
| 26 | TRAV16_02 | CTTTCCACCTGAAGAAACCATTTGCTCAAGAGGAAGACTC |
| 27 | TRAV17_00 | AAGAGGATCCTCAGGCCTTGAGCATCCAGGAGGGTGAAAA |
| 28 | TRAV17_01 | GACAAAATTCAGGTAGAGGCCTTGTCCACCTAATTTTAAT |
| 29 | TRAV17_02 | AGTGGAAGATTAAGAGTCACGCTTGACACTTCCAAGAAAA |
| 30 | TRAV17_03 | CACGGCTTCCCGGGCAGCAGACACTGCTTCTTACTTCTGT |
| 31 | TRAV18_00 | GCCCAGTTACCCTCCCTGAGAGGGCAGCTCTGACATTAAA |
| 32 | TRAV18_01 | TGTCCAGTATCTAAACAAAGAGCCTGAGCTCCTCCTGAAA |
| 33 | TRAV18_02 | TCCTATCAAGAGTGACAGTTCCTTCCACCTGGAGAAGCCC |
| 34 | TRAV19_00 | CTCAGAAGGTAACTCAAGCGCAGACTGAAATTTCTGTGGT |
| 35 | TRAV19_01 | GAAGGAGGATGTGACCTTGGACTGTGTGTATGAAACCCGT |
| 36 | TRAV19_02 | TCTGGTACAAGCAACCACCAAGTGGAGAATTGGTTTTCCT |
| 37 | TRAV20_00 | TATAGGCAAGATCCTGGGAAAGGCCCTGAATTCCTCTTCA |
| 38 | TRAV20_01 | CACAGCCCCTAAACCTGAAGACTCAGCCACTTATCTCTGT |
| 39 | TRAV20_02 | CTGCAGTTACACAGTCAGCGGTTTAAGAGGGCTGTTCTGG |
| 40 | TRAV21_00 | GGTTCTCAACTGCAGTTTCACTGATAGCGCTATTTACAAC |
| 41 | TRAV21_01 | AGTGGTTTAGGCAGGACCCTGGGAAAGGTCTCACATCTCT |
| 42 | TRAV21_02 | ATGCCTCGCTGGATAAATCATCAGGACGTAGTACTTTATA |
| 43 | TRAV22_00 | TTTTCTGACTCTGTGAACAATTTGCAGTGGTTTCATCAAA |
| 44 | TRAV22_01 | TCCAGACCTGATTCTCCAGGAGGGAGCCAATTCCACGCTG |
| 45 | TRAV22_02 | TTTTTCTGACTCTGTGAACAATTTGCAGTGGTTTCATCAA |
| 46 | TRAV22_03 | CAGAATGGAAGATTAAGCGCCACGACTGTCGCTACGGAAC |
| 47 | TRAV22_04 | GCCACGACTGTCGCTACGGAACGCTACAGCTTATTGTACA |
| 48 | TRAV22_05 | TCCCAGACCACAGACTCAGGCGTTTATTTCTGTGCTGTGG |
| 49 | TRAV23/DV6_00 | TATAAACTGTGCTTATGAGAACACTGCGTTTGACTACTTT |
| 50 | TRAV23/DV6_01 | CATGGTACCAACAATTCCCTGGGAAAGGCCCTGCATTATT |
| 51 | TRAV23/DV6_02 | CAAAGTCCTCAATCTTTGATAGTCCAGAAAGGAGGGATTT |
| 52 | TRAV24_00 | TACTGAACGTGGAACAAAGTCCTCAGTCACTGCATGTTCA |
| 53 | TRAV24_01 | GGAGACAGCACCAATTTCACCTGCAGCTTCCCTTCCAGCA |
| 54 | TRAV24_02 | ATGCCTTACACTGGTACAGATGGGAAACTGCAAAAAGCCC |
| 55 | TRAV25_00 | AGAAGGAGAGGACTTCACCACGTACTGCAATTCCTCAACT |
| 56 | TRAV25_01 | TTTAAGCAATATACAGTGGTATAAGCAAAGGCCTGGTGGA |
| 57 | TRAV25_02 | ATACAGTTAGTGAAGAGTGGAGAAGTGAAGAAGCAGAAAA |
| 58 | TRAV26-1_00 | CCATGGATTGCGCTGAAGGAAGAGCTGCAAACCTGCCTTG |
| 59 | TRAV26-1_01 | GCCTTGTAATCACTCTACCATCAGTGGAAATGAGTATGTG |
| 60 | TRAV26-1_02 | AAACAATGAAACCAATGAAATGGCCTCTCTGATCATCACA |
| 61 | TRAV26-2_00 | TCGACAGCTTCCCTCCCAGGGTCCAGAGTACGTGATTCAT |
| 62 | TRAV26-2_01 | TCTTACAAGCAATGTGAACAACAGAATGGCCTCTCTGGCA |
| 63 | TRAV26-2_02 | CCTTGATCCTGCACCGTGCTACCTTGAGAGATGCTGCTGT |
| 64 | TRAV27_00 | CCCAGCTGCTGGAGCAGAGCCCTCAGTTTCTAAGCATCCA |
| 65 | TRAV27_01 | AAGGTCCTGTCCTCCTGGTGACAGTAGTTACGGGTGGAGA |
| 66 | TRAV27_02 | GTTTGGTGATGCAAGAAAGGACAGTTCTCTCCACATCACT |
| 67 | TRAV29/DV5_00 | ACCCTGCTGAAGGTCCTACATTCCTGATATCTATAAGTTC |
| 68 | TRAV29/DV5_01 | ACCATCCCTGAGCGTCCAGGAAGGAAGAATTTCTATTCTG |
| 69 | TRAV29/DV5_02 | TGCACATTGTGCCCTCCCAGCCTGGAGACTCTGCAGTGTA |
| 70 | TRAV2_00 | GGAGCTGTGGTGGAAATCTTCTGTAATCACTCTGTGTCCA |
| 71 | TRAV2_01 | CGATACAACATGACCTATGAACGGTTCTCTTCATCGCTGC |
| 72 | TRAV2_02 | GCTCATCCTCCAGGTGCGGGAGGCAGATGCTGCTGTTTAC |
| 73 | TRAV30_00 | AGATGCTGTCATCAACTGCAGTTCCTCCAAGGCTTTATAT |
| 74 | TRAV30_01 | TGTACACTGGTACAGGCAGAAGCATGGTGAAGCACCCGTC |
| 75 | TRAV30_02 | AAGCTCCCTGTACCTTACGGCCTCCCAGCTCAGTTACTCA |
| 76 | TRAV34_00 | TCAGTCCTTGATCGTCCAAGAGGGAAAGAATCTCACCATA |
| 77 | TRAV34_01 | TAAACTGCACGTCATCAAAGACGTTATATGGCTTATACTG |
| 78 | TRAV34_02 | CTTATCTTCTTGATGATGCTACAGAAAGGTGGGGAAGAGA |
| 79 | TRAV35_00 | GAAGATGTCTCCATGAACTGCACTTCTTCAAGCATATTTA |
| 80 | TRAV35_01 | TGACCTCAAATGGAAGACTGACTGCTCAGTTTGGTATAAC |
| 81 | TRAV35_02 | AAAGGACAGCTTCCTGAATATCTCAGCATCCATACCTAGT |
| 82 | TRAV36/DV7_00 | AAGACAAGGTGGTACAAAGCCCTCTATCTCTGGTTGTCCA |
| 83 | TRAV36/DV7_01 | CTAACTTTCGAAGCCTACTATGGTACAAGCAGGAAAAGAA |
| 84 | TRAV36/DV7_02 | GGGAGACACCGTAACTCTCAATTGCAGTTATGAAGTGACT |
| 85 | TRAV38-2/DV8_00 | ATTTATTCTGGTACAAGCAGCCTCCCAGCAGGCAGATGAT |
| 86 | TRAV38-2/DV8_01 | TATTCGCCAAGAAGCTTATAAGCAACAGAATGCAACAGAG |
| 87 | TRAV38-2/DV8_02 | TTTCTCTGTGAACTTCCAGAAAGCAGCCAAATCCTTCAGT |
| 88 | TRAV39_00 | AACAAAACCCTCTGTTCCTGAGCATGCAGGAGGGAAAAAA |
| 89 | TRAV3_00 | CTCAGTCAGTGGCTCAGCCGGAAGATCAGGTCAACGTTGC |
| 90 | TRAV3_01 | AGGGAATCCTCTGACTGTGAAATGCACCTATTCAGTCTCT |
| 91 | TRAV3_02 | AGGCCTCCAGTTCCTTCTGAAATACATCACAGGGGATAAC |
| 92 | TRAV40_00 | GCAATTCAGTCAAGCAGACGGGCCAAATAACCGTCTCGGA |
| 93 | TRAV40_01 | GGGAGCATCTGTGACTATGAACTGCACATACACATCCACG |
| 94 | TRAV40_02 | AACCTCTGCAGCTTCTTCAGAGAGAGACAATGGAAAACAG |
| 95 | TRAV41_00 | ATTTATCACAATCAACTGCAGTTACTCGGTAGGAATAAGT |
| 96 | TRAV41_01 | AGTGCCTTACACTGGCTGCAACAGCATCCAGGAGGAGGCA |
| 97 | TRAV41_02 | TATGCTGAGCTCAGGGAAGAAGAAGCATGGAAGATTAATT |
| 98 | TRAV4_00 | ATCTCCATGGACTCATATGAAGGACAAGAAGTGAACATAA |
| 99 | TRAV4_01 | AAATGATTATATCACGTGGTACCAACAGTTTCCCAGCCAA |
| 100 | TRAV4_02 | GTTACAAACGAAGTGGCCTCCCTGTTTATCCCTGCCGACA |
| 101 | TRAV5_00 | CACAGACAGCTCCTCCACCTACTTATACTGGTATAAGCAA |
| 102 | TRAV5_01 | TCTGTCTCTGCGCATTGCAGACACCCAGACTGGGGACTCA |
| 103 | TRAV6_00 | AAATATGGACATGAAACAAGACCAAAGACTCACTGTTCTA |
| 104 | TRAV6_01 | ACTATACAAACTATTCTCCAGCATACTTACAGTGGTACCG |
| 105 | TRAV6_02 | GAAAGAAAGACTGAAGGTCACCTTTGATACCACCCTTAAA |
| 106 | TRAV6_03 | AGAGTTTGTTTCATATCACAGCCTCCCAGCCTGCAGACTC |
| 107 | TRAV7_00 | AATACAGGGATGGGTCCCAAACACCTATTATCCATGTATT |
| 108 | TRAV7_01 | AAATGCTACATTACTGAAGAATGGAAGCAGCTTGTACATT |
| 109 | TRAV7_02 | ACAGCCGTGCAGCCTGAAGATTCAGCCACCTATTTCTGTG |
| 110 | TRAV8-1_00 | CCAGTCTGTGAGCCAGCATAACCACCACGTAATTCTCTCT |
| 111 | TRAV8-1_01 | GTTGGGATGCAACTATTCCTATGGTGGAACTGTTAATCTC |
| 112 | TRAV8-1_02 | TATGTCCAGTACCCTGGTCAACACCTTCAGCTTCTCCTCA |
| 113 | TRAV8-2/4/6_00 | ACTCCAGCTTCTCCTGAAGTACACATCAGCGGCCACCCTG |
| 114 | TRAV8-2/4/6_01 | GGTGCAACTACTCATCGTCTGTTTCAGTGTATCTCTTCTG |
| 115 | TRAV9-1_00 | GATTCAGTGGTCCAGACAGAAGGCCAAGTGCTCCCCTCTG |
| 116 | TRAV9-1_01 | ACTGCTCCTATGAAACCACACAGTACCCTTCCCTTTTTTG |
| 117 | TRAV9-2_00 | AGAGGCCTTCCTGACTATAAACTGCACGTACACAGCCACA |
| 118 | TRAV9-2_01 | AGATTCAGTGACCCAGATGGAAGGGCCAGTGACTCTCTCA |
| 119 | TRBC1_00 | CGACCACGTGGAGCTGAGCTGGTGGGTGAATGGGAAGGAG |
| 120 | TRBC1_01 | CAAGGAGCAGCCCGCCCTCAATGACTCCAGATACTGCCTG |
| 121 | TRBC1_02 | AGGGGTCCTGTCTGCCACCATCCTCTATGAGATCCTGCTA |
| 122 | TRBJ1-1_00 | GAAGCTTTCTTTGGACAAGGCACCAGACTCACAGTTGTAG |
| 123 | TRBJ2-2_00 | GCTGTTTTTTGGAGAAGGCTCTAGGCTGACCGTACTGGAG |
| 124 | TRBV10-3_00 | ACAAGGTCACAGAGACAGGAACACCAGTGACTCTGAGATG |
| 125 | TRBV11-1/2/3_00 | TCTGCAGAGAGGCTCAAAGGAGTAGACTCCACTCTCAAGA |
| 126 | TRBV11-1/2/3_01 | GCAGAGAGGCTCAAAGGAGTAGACTCCACTCTCAAGATCC |
| 127 | TRBV12-3/4_00 | GCGGGGACTGGAGTTGCTCATTTACTTTAACAACAACGTT |
| 128 | TRBV12-3/4_01 | CTTTAACAACAACGTTCCGATAGATGATTCAGGGATGCCC |
| 129 | TRBV12-3/4_02 | GATTCTCAGCTAAGATGCCTAATGCATCATTCTCCACTCT |
| 130 | TRBV12-5_00 | ATGATGCAAGGACTGGAGTTGCTGGCTTACTTCCGCAACC |
| 131 | TRBV12-5_01 | GGATCGATTCTCAGCAGAGATGCCTGATGCAACTTTAGCC |
| 132 | TRBV13_00 | CACTCTGAAATGCTATCCTATCCCTAGACACGACACTGTC |
| 133 | TRBV13_01 | CGATTCTCAGCTCAACAGTTCAGTGACTATCATTCTGAAC |
| 134 | TRBV14_00 | GAAAGAGTCTAAACAGGATGAGTCCGGTATGCCCAACAAT |
| 135 | TRBV14_01 | ATCGATTCTTAGCTGAAAGGACTGGAGGGACGTATTCTAC |
| 136 | TRBV14_02 | CTGAAGGTGCAGCCTGCAGAACTGGAGGATTCTGGAGTTT |
| 137 | TRBV15_00 | TGGTCATCCAGAACCCAAGATACCAGGTTACCCAGTTTGG |
| 138 | TRBV15_01 | TGTTCTCAGACTTTGAACCATAACGTCATGTACTGGTACC |
| 139 | TRBV15_02 | AAAGCTGCTGTTCCACTACTATGACAAAGATTTTAACAAT |
| 140 | TRBV16_00 | TTCTCCTGGTGAAGAAGTCGCCCAGACTCCAAAACATCTT |
| 141 | TRBV18_00 | CACAGTCATGTTTACTGGTATCGGCAGCTCCCAGAGGAAG |
| 142 | TRBV18_01 | TCTCTCAAATGCCGGCGTCATGCAGAACCCAAGACACCTG |
| 143 | TRBV18_02 | CAAGACTGAGATGCAGCCCAATGAAAGGACACAGTCATGT |
| 144 | TRBV18_03 | GGCAGCTCCCAGAGGAAGGTCTGAAATTCATGGTTTATCT |
| 145 | TRBV18_04 | ATATCATAGATGAGTCAGGAATGCCAAAGGAACGATTTTC |
| 146 | TRBV18_05 | GGGCCCCAGCATCCTGAGGATCCAGCAGGTAGTGCGAGGA |
| 147 | TRBV19_00 | TACCTGTTCAGAAAGGAAGGACAGAATGTGACCCTGAGTT |
| 148 | TRBV19_01 | GCAAGGGCTGAGATTGATCTACTACTCACAGATAGTAAAT |
| 149 | TRBV19_02 | CAGCGTCTCTCGGGAGAAGAAGGAATCCTTTCCTCTCACT |
| 150 | TRBV20-1_00 | GGGTTATCTGTAAGAGTGGAACCTCTGTGAAGATCGAGTG |
| 151 | TRBV20-1_01 | GAAGGACAAGTTTCTCATCAACCATGCAAGCCTGACCTTG |
| 152 | TRBV20-1_02 | GTCCACTCTGACAGTGACCAGTGCCCATCCTGAAGACAGC |
| 153 | TRBV24-1_00 | TCACAAAGACAGGAAAGAGGATTATGCTGGAATGTTCTCA |
| 154 | TRBV24-1_01 | AGGAGAGATCTCTGATGGATACAGTGTCTCTCGACAGGCA |
| 155 | TRBV24-1_02 | GCTAAATTCTCCCTGTCCCTAGAGTCTGCCATCCCCAACC |
| 156 | TRBV25-1_00 | TGGTATCAACAAGATCCAGGAATGGAACTACACCTCATCC |
| 157 | TRBV25-1_01 | CACTATTCCTATGGAGTTAATTCCACAGAGAAGGGAGATC |
| 158 | TRBV25-1_02 | TGACCCTGGAGTCTGCCAGGCCCTCACATACCTCTCAGTA |
| 159 | TRBV27_00 | CAGTGACTGGAAAGAAGTTAACAGTGACTTGTTCTCAGAA |
| 160 | TRBV27_01 | AAGACCCAGGGCTGGGCTTAAGGCAGATCTACTATTCAAT |
| 161 | TRBV27_02 | GAATGTTGAGGTGACTGATAAGGGAGATGTTCCTGAAGGG |
| 162 | TRBV28_00 | GCCTCGTAGATGTGAAAGTAACCCAGAGCTCGAGATATCT |
| 163 | TRBV28_01 | AAGTTTTTCTGGAATGTGTCCAGGATATGGACCATGAAAA |
| 164 | TRBV2_00 | ACAGATGGGACAGGAAGTGATCTTGCGCTGTGTCCCCATC |
| 165 | TRBV2_01 | CAGAACCTGAAGTCACCCAGACTCCCAGCCATCAGGTCAC |
| 166 | TRBV2_02 | ACAGATGGGACAGGAAGTGATCTTGCGCTGTGTCCCCATC |
| 167 | TRBV2_03 | TATTCGATGATCAATTCTCAGTTGAAAGGCCTGATGGATC |
| 168 | TRBV2_04 | GATCCGGTCCACAAAGCTGGAGGACTCAGCCATGTACTTC |
| 169 | TRBV3-1_00 | GATGGGAAACGACAAGTCCATTAAATGTGAACAAAATCTG |
| 170 | TRBV30_00 | GGGCAGCCCGCTCTCTCTGGAGTGCACTGTGGAGGGAACA |
| 171 | TRBV30_01 | GGGCCTCCAGCTGCTCTTCTACTCCGTTGGTATTGGCCAG |
| 172 | TRBV30_02 | TGACTCTGGCTTCTATCTCTGTGCCTGGAGTGTCACACTG |
| 173 | TRBV4-2_00 | TGGAAACGGGAGTTACGCAGACACCAAGACACCTGGTCAT |
| 174 | TRBV4-2_01 | TAACGCTATGTATTGGTACAAGCAAAGTGCTAAGAAGCCA |
| 175 | TRBV4-2_02 | AACAACAGTGTGCCAAGTCGCTTCTCACCTGAATGCCCCA |
| 176 | TRBV5-1_00 | GTAAAGGCTGGAGTCACTCAAACTCCAAGATATCTGATCA |
| 177 | TRBV5-1_01 | GGACAGGGCCTTCAGTTCCTCTTTGAATACTTCAGTGAGA |
| 178 | TRBV5-4_00 | TCAGTATTATAGGGAGGAAGAGAATGGCAGAGGAAACTTC |
| 179 | TRBV5-6_00 | TATCTTTCAGTATTATGAGGAGGAAGAGAGACAGAGAGGC |
| 180 | TRBV5-8_00 | GTCTGGGCCTCCAGTTCCTCCTTTGGTATGACGAGGGTGA |
| 181 | TRBV6-4_00 | GATCCTGGCAGCAGGACGGCGCATGACACTGAGATGTACC |
| 182 | TRBV6-4_01 | GGCTCATCCATTATTCAAATACTGCAGGTACCACTGGCAA |
| 183 | TRBV6-4_02 | CTCACGTTGGCGTCTGCTGTACCCTCTCAGACATCTGTGT |
| 184 | TRBV6-8_00 | ACTCAGCTGCTGCTGGTACTACTGACAAAGAAGTCCCCAA |
| 185 | TRBV7-2_00 | TTTAATTTACTTCCAAGGCAACAGTGCACCAGACAAATCA |
| 186 | TRBV7-3_00 | TCTAATTTACTTCCAAGGCACGGGTGCGGCAGATGACTCA |
| 187 | TRBV7-9_00 | AACTTTCAGGTGTGATCCAATTTCTGAACACAACCGCCTT |
| 188 | TRBV9_00 | CACCTGATCACAGCAACTGGACAGCGAGTGACGCTGAGAT |
| 189 | TRBV9_01 | GTCTGGAGACCTCTCTGTGTACTGGTACCAACAGAGCCTG |
| 190 | TRBV9_02 | AACATTCTTGAACGATTCTCCGCACAACAGTTCCCTGACT |
| 191 | TRDC_00 | AAATGTCGCTTGTCTGGTGAAGGAATTCTACCCCAAGGAT |
| 192 | TRDC_01 | AGTTCATACCGAGAAGGTGAACATGATGTCCCTCACAGTG |
| 193 | TRDV1_00 | GTATCCATGCCAGTGAGGAAAGCAGTCACCCTGAACTGCC |
| 194 | TRDV1_01 | TCGCTATTCTGTCAACTTCAAGAAAGCAGCGAAATCCGTC |
| 195 | TRDV1_02 | GCCTTAACCATTTCAGCCTTACAGCTAGAAGATTCAGCAA |
| 196 | TRDV2_00 | CATCTATGGCCCTGGTTTCAAAGACAATTTCCAAGGTGAC |
| 197 | TRDV2_01 | CCCTGCCACCCTCAGGTGCTCCATGAAAGGAGAAGCGATC |
| 198 | TRDV2_02 | TAACTACTATATCAACTGGTACAGGAAGACCCAAGGTAAC |
| 199 | TRDV3_00 | TTTATGGGGATAACAGCAGATCAGAAGGTGCAGATTTTAC |
| 200 | TRDV3_01 | GTGGCGAGTGGCAGTGAGGTGGTACTGCTCTGCACTTACG |
| 201 | TRDV3_02 | CTCTCCAGTAAGGACTGAAGACAGTGCCACTTACTACTGT |
| 202 | TRGV2/4_00 | ACTTGTGATCTTGCTGAAGGAAGTAACGGCTACATCCACT |
| 203 | TRGV2/4_01 | GAAGCACAAGGAAGAACTTGAGAATGATACTGCGAAATCT |
| 204 | TRGV3/5_00 | TCAGTCCAGGAAAGTATTATACTCATACACCCAGGAGGTG |
| 205 | TRGV3/5_01 | CAGGAAAGTATTATACTCATACACCCAGGAGGTGGAGCTG |
| 206 | TRGV8_00 | ATACTTATGCAAGCACAGGGAAGAGCCTTAAATTTATACT |
| 207 | TRGV9_00 | GTGTGTATATGGTGCAGGTCACCTAGAGCAACCTCAAATT |
| 208 | TRGV9_01 | CAAAAACAGCCCGCCTGGAATGTGTGGTGTCTGGAATAAA |

| **Supplementary Table 5:** Predesigned Xenium Human Multi-Tissue and Cancer panel gene list (377 genes). | | | | | |
| --- | --- | --- | --- | --- | --- |
| Gene name | | | | | |
| ABCC11 | C15orf48 | CD83 | CYP3A4 | FSTL3 | INS |
| ACE2 | C15orf162 | CD86 | CYP4B1 | FXYD2 | IRF8 |
| ACKR1 | C15orf194 | CD8A | CYTIP | GATA2 | KCNK3 |
| ACTA2 | C20orf85 | CD93 | DERL3 | GATM | KCNMA1 |
| ACTG2 | C5orf46 | CDH16 | DES | GCG | KIT |
| ADAM28 | C6orf118 | CDK1 | DIRAS3 | GDF15 | KLK11 |
| ADAMTS1 | C7 | CENPF | DMBT1 | GEM | KLRB1 |
| ADGRE1 | CA4 | CFAP53 | DNAAF1 | GHRL | KLRC1 |
| ADGRL4 | CAPN8 | CFB | DNASE1L3 | GKN2 | KLRD1 |
| ADH1C | CAV1 | CFHR1 | DPEP1 | GLIPR1 | KNG1 |
| ADH4 | CAVIN1 | CFHR3 | DPT | GLYATL1 | KRT20 |
| ADIPOQ | CAVIN2 | CFTR | DST | GNG11 | KRT7 |
| AGER | CCDC39 | CHGA | DUSP2 | GNLY | LAG3 |
| AGR3 | CCDC78 | CLCA1 | ECSCR | GPC1 | LAMP3 |
| AHSP | CCL19 | CLCA2 | EDN1 | GPC3 | LGI4 |
| AIF1 | CCL27 | CLEC10A | EDNRB | GPR183 | LGR5 |
| ALAS2 | CCL5 | CLEC14A | EGFL7 | GPRC5A | LIF |
| ALDH1A3 | CCNB2 | CLEC4E | EGFR | GPX2 | LILRA4 |
| AMY2A | CCR2 | CLECL1 | EHF | GYPA | LILRA5 |
| ANGPT2 | CCR7 | CLIC6 | ELF5 | GYPB | LILRB2 |
| ANPEP | CD14 | CNN1 | EPCAM | GZMA | LILRB4 |
| APCDD1 | CD163 | COCH | ERBB2 | GZMB | LPL |
| APOA5 | CD19 | COL17A1 | ERG | GZMK | LTBP2 |
| APOBEC3A | CD1A | COL5A2 | ESR1 | HAMP | LY6D |
| APOLD1 | CD1C | CPA3 | FAS | HAVCR2 | LY86 |
| AQP2 | CD1E | CRHBP | FBLN1 | HEMGN | LYVE1 |
| AQP3 | CD2 | CRISPLD2 | FBN1 | HEPACAM2 | MALL |
| AQP8 | CD247 | CSF2RA | FCER1A | HES4 | MAMDC2 |
| AQP9 | CD27 | CSF3 | FCGR1A | HIGD1B | MARCO |
| AR | CD274 | CTLA4 | FCGR3A | HLA-DQB2 | MCEMP1 |
| ARFGEF3 | CD28 | CTSG | FCN1 | HMGCS2 | MCF2L |
| ASCL1 | CD300E | CTSK | FCN2 | HPGDS | MDM2 |
| ASCL3 | CD34 | CXCL10 | FGFBP1 | HPX | MEDAG |
| ASPN | CD3D | CXCL2 | FGFBP2 | IGF1 | MEF2C |
| BAMBI | CD3E | CXCL6 | FGL2 | IGSF6 | MEST |
| BANK1 | CD4 | CXCL9 | FHL2 | IL1R2 | MET |
| BASP1 | CD5L | CXCR4 | FKBP11 | IL1RL1 | MFAP5 |
| BBOX1 | CD68 | CYP1A1 | FOXA1 | IL2RA | MKI67 |
| BCL2L11 | CD69 | CYP2A7 | FOXI1 | IL3RA | MLANA |
| BMX | CD70 | CYP2B6 | FOXJ1 | IL7R | MLPH |
| BTNL9 | CD79A | CYP2F1 | FOXP3 | INMT | MMRN1 |
|  |  |  |  |  |  |
|  |  |  |  |  |  |
|  |  |  |  |  |  |
| Gene name | | | | | |
| MMRN2 | PRF1 | SNTN | UPK3B |  |  |
| MNDA | PRG4 | SOX17 | VCAN |  |  |
| MPEG1 | PROX1 | SOX18 | VSIG4 |  |  |
| MRC1 | PTGDS | SOX2 | VWA5A |  |  |
| MS4A1 | PTN | SPDEF | VWF |  |  |
| MS4A2 | PTPRC | SPI1 |  |  |  |
| MS4A4A | PVALB | SPIB |  |  |  |
| MS4A6A | RAMP2 | SRPX |  |  |  |
| MTRNR2L11 | RAPGEF3 | SST |  |  |  |
| MYBPC1 | RBP5 | STC1 |  |  |  |
| MYC | RERGL | STC2 |  |  |  |
| MYH11 | RETN | STEAP4 |  |  |  |
| MYLK | RGS16 | TAC1 |  |  |  |
| MZB1 | RIDA | TAT |  |  |  |
| NAT8 | RND1 | TBX3 |  |  |  |
| NKG7 | RTKN2 | TCF15 |  |  |  |
| NPDC1 | S100A1 | TCF4 |  |  |  |
| NTN4 | S100A12 | TCIM |  |  |  |
| OGN | SCGB2A1 | TCL1A |  |  |  |
| OPRPN | SCGN | TENT5C |  |  |  |
| PCNA | SELE | TFF2 |  |  |  |
| PCOLCE | SELL | TFPI |  |  |  |
| PCP4 | SEMA3C | THAP2 |  |  |  |
| PCSK2 | SERPINB2 | THBS2 |  |  |  |
| PDCD1 | SERPINB3 | THY1 |  |  |  |
| PDGFRA | SERPINB9 | TIMP4 |  |  |  |
| PDGFRB | SFRP2 | TM4SF18 |  |  |  |
| PDPN | SFRP4 | TM4SF4 |  |  |  |
| PEBP4 | SFTA2 | TMC5 |  |  |  |
| PECAM1 | SH2D3C | TMEM100 |  |  |  |
| PGR | SLAMF1 | TMEM174 |  |  |  |
| PLA2G7 | SLAMF7 | TMEM52B |  |  |  |
| PLAC9 | SLC18A2 | TNC |  |  |  |
| PLCG2 | SLC22A8 | TNFRSF13B |  |  |  |
| PLD4 | SLC26A2 | TNFRSF17 |  |  |  |
| PLIN4 | SLC26A3 | TNFRSF9 |  |  |  |
| PMP22 | SLC4A1 | TOP2A |  |  |  |
| PPARG | SMIM24 | TRAC |  |  |  |
| PPP1R1A | SMYD2 | TREM2 |  |  |  |
| PPP1R1B | SNAI1 | TSPAN19 |  |  |  |
| PPY | SNCA | UBE2C |  |  |  |
| PRDM1 | SNCG | UMOD |  |  |  |

**Supplementary Table 6**: Correlation of Lymphotrack, RNAseq, ISS and Xenium in TRBV gene detection.

|  | Adenocarcinoma | Squamous cell carcinoma |
| --- | --- | --- |
| Comparison | Spearman (r) | Spearman (r) |
| Lymphotrack vs RNA seq | 0.4786 | -0.1476 |
| Xenium vs Lymphotrack | 0.6926 | 0.1742 |
| Xenium vs RNA seq | 0.5885 | 0.4952 |
| Xenium vs ISS | 0.6209 | 0.3092 |

**Supplementary Table 7**: Raw counts of TRBV genes detected with Lymphotrack, RNAseq, ISS and Xenium in the adenocarcinoma lung biopsy. Marked with x are the TRBV genes that were not present in the results of each method.

|  | Lymphotrack | RNA seq | ISS | Xenium |
| --- | --- | --- | --- | --- |
| TRBV10-3 | 338 | 0 | 1658 | 2141 |
| TRBV11-1/-2/-3 | 2756 | 14 | 504 | 2141 |
| TRBV12-3/-4 | 4618 | 0 | 924 | 1845 |
| TRBV12-5 | 0 | 0 | 470 | 200 |
| TRBV13 | 1050 | 0 | 575 | 2378 |
| TRBV14 | 8786 | 0 | 1656 | 2015 |
| TRBV15 | 1576 | 0 | 1618 | 614 |
| TRBV16 | 0 | 0 | 1037 | 66 |
| TRBV18 | 2271 | 0 | 3584 | 8683 |
| TRBV19 | 3727 | 292 | 8653 | 40316 |
| TRBV2 | 0 | 8 | 53 | 831 |
| TRBV24-1 | 2085 | 133 | 5516 | 11363 |
| TRBV25-1 | 300 | 0 | 894 | 662 |
| TRBV27 | 694 | 0 | 1112 | 10969 |
| TRBV28 | 2798 | 164 | 4775 | 12932 |
| TRBV30 | 33061 | 290 | 4340 | 12871 |
| TRBV3-1 | 14420 | 22 | 398 | 2238 |
| TRBV4-2 | 2532 | 142 | 1201 | 28398 |
| TRBV5-1 | 12223 | 62 | 826 | 6504 |
| TRBV5-4 | x | x | x | 1906 |
| TRBV5-6 | x | x | x | 2842 |
| TRBV5-8 | 0 | 0 | 136 | 19 |
| TRBV6-4 | 459 | x | x | 361 |
| TRBV6-8 | 0 | 0 | 746 | 26 |
| TRBV7-2 | 1745 | 0 | 1466 | 4288 |
| TRBV7-3 | 0 | 21 | 1068 | 306 |
| TRBV7-9 | 576 | 0 | 1501 | 2275 |
| TRBV9 | 0 | 7 | 226 | 823 |

**Supplementary Table 8**: Raw counts of TRBV genes detected with Lymphotrack, RNAseq, ISS and Xenium in the squamous cell carcinoma lung biopsy. Marked with x are the TRBV genes that were not present in the results of each method.

|  | Lymphotrack | RNA seq | ISS | Xenium |
| --- | --- | --- | --- | --- |
| TRBV10-3 | 406 | 0 | 1842 | 563 |
| TRBV11-1/-2/-3 | 1551 | 28 | 412 | 785 |
| TRBV12-3/-4 | 16450 | 0 | 797 | 672 |
| TRBV12-5 | 0 | 0 | 1477 | 80 |
| TRBV13 | 453 | 0 | 945 | 248 |
| TRBV14 | 7909 | 0 | 4094 | 665 |
| TRBV15 | 8913 | 0 | 1883 | 109 |
| TRBV16 | 0 | 0 | 716 | 68 |
| TRBV18 | 6569 | 0 | 5425 | 2695 |
| TRBV19 | 0 | 59 | 4388 | 2248 |
| TRBV2 | 2067 | 31 | 41 | 1241 |
| TRBV24-1 | 0 | 7 | 1732 | 470 |
| TRBV25-1 | 4603 | 0 | 690 | 131 |
| TRBV27 | 0 | 1 | 1136 | 1892 |
| TRBV28 | 0 | 33 | 7654 | 904 |
| TRBV30 | 0 | 18 | 4571 | 787 |
| TRBV3-1 | 0 | 22 | 403 | 428 |
| TRBV4-2 | 389 | 47 | 570 | 2639 |
| TRBV5-1 | 1428 | 55 | 321 | 1221 |
| TRBV5-4 | x | x | x | 1215 |
| TRBV5-6 | x | x | x | 503 |
| TRBV5-8 | 0 | 0 | 226 | 38 |
| TRBV6-4 | 1876 | x | x | 395 |
| TRBV6-8 | 0 | 0 | 271 | 11 |
| TRBV7-2 | 0 | 0 | 3755 | 3053 |
| TRBV7-3 | 0 | 32 | 1341 | 128 |
| TRBV7-9 | 0 | 0 | 56 | 450 |
| TRBV9 | 1439 | 38 | 890 | 1089 |

| **Supplementary Table 9:** Top 15 differentially expressed genes in identified cell clusters. | |
| --- | --- |
| Benign alveolar cells_1 | CXCL2, RND1, GPRC5A, LAMP3, NTN4, TCIM, CYP4B1, CAPN8, AGR3, TFPI, MALL, CFTR, MLPH, CYP2B6, FOLR1 |
| Benign alveolar cells_2 | CXCL2, LAMP3, RND1, CAPN8, NTN4, TCIM, TFPI, CYP4B1, AGR3, GPRC5A, CYP2B6, MLPH, CFTR,MALL, SFTA2 |
| CAFs | COL5A2, VCAN, PDGFRB, THY1, PCOLCE, FBN1, FBLN1, SERPINB9, LTBP2, THBS2, MYLK, PDGFRA, CRISPLD2, CTSK, GEM |
| Cancer_1 | MKI67, TOP2A, CENPF, UBE2C, CFB, KRT7, BASP1, CDK1, CCNB2, SOX2, EPCAM, TCIM, MLPH, GPRC5A, ACTG2 |
| Cancer_2 | MKI67, TOP2A, CENPF, CFB, UBE2C, KRT7, EPCAM, BASP1, TCIM, CDK1, CCNB2, SOX2, GPRC5A, TACSTD2, FOLR1 |
| Cancer_3 | CFB, KRT7, TCIM, SERPINB9, NECTIN4, MLPH, EPCAM, GPRC5A, TMC5, BASP1, TACSTD2, AQP3, ACTG2, STEAP4, FOLR1 |
| Cancer_4 | CFB, KRT7, TCIM, MLPH, EPCAM, BASP1, TMC5, SOX2, FOLR1, KLK11, STEAP4, NECTIN4, GPRC5A, PCNA, CYP4B1 |
| Cancer_5 | CFB, TCIM, KRT7, SERPINB9, MLPH, EPCAM, TMC5, BASP1, NECTIN4, GPRC5A, FOLR1, STEAP4, SOX2, PCNA, TACSTD2 |
| Cancer_6 | CFB, KRT7, TCIM, MLPH, EPCAM, PCNA, BASP1, TMC5, NECTIN4, TACSTD2, GPRC5A, SERPINB9, FOLR1, STEAP4, ERBB2 |
| Cancer_7 | VCAN, THY1, PDGFRB, CTSK, COL5A2, FBLN1, CFB, CRISPLD2, HLA-A, TCIM, TNC, BASP1, RGS16, IDO1,MEST |
| Cancer_8 | KRT7, CFB, TCIM, MLPH, NECTIN4, ACTG2, EPCAM, TMC5, BASP1, STEAP4, GPRC5A, FOLR1,AQP3,TACSTD2, SOX2 |
| Cancer_9 | CFB, KRT7, PCNA, TCIM, MLPH, EPCAM, TMC5, BASP1, ACTG2, FOLR1, NECTIN4, STEAP4, TACSTD2, GPRC5A, AQP3 |
| Cancer_10 | CFB, KRT7, MLPH, TCIM, BASP1, TMC5, EPCAM, PCNA, MDM2, NECTIN4, STEAP4, SOX2, ERBB2,FOLR1, ACTG2 |
| CD4 activated | TRAC, TRBC1, IL7R, CD2, CXCR4, PTPRC, CD247, CD28, CYTIP, CCR7, CTLA4, CD3D, CD4, CD3E, ACTG2 |
| CD4 TLS associated T cells_1 | SERPINB9, IL7R, CXCR4, CCR7, TRAC, TRBC1, PTPRC, CD28, SELL, CD4, CD2, CYTIP, CD247, CD27, SLAMF1 |
| CD4 TLS associated T cells_2 | IL7R, TRAC, CXCR4, CCR7, TRBC1, CD28, CD247, SELL, MS4A1, CD2, SLAMF1, CTLA4, PTGDS, CD27, GZMK |
| CD8 T cells_1 | CCL5, PTPRC, TRAC, TRBC1, CD3E, SERPINB9, CD3D, GZMB, CD2, CD247, HLA-A, GZMA, CYTIP, CD8A, KLRC1 |
| CD8 T cells_2 | CCL5, PTPRC, TRAC, TRBC1, CD3D, CD3E, SERPINB9, HLA-A, CD2, GZMB, GZMA, CD247, KLRC1, CD8A, CFB |
| CD8 T cells_3 | CCL5, PTPRC, TRAC, TRBC1, GZMB, CD3E, CD3D, CD2, GZMA, CD8A, CD247, GNLY, KLRC1, LAG3, CYTIP |
| CD8 T cells_4 | CCL5, TRAC, PTPRC, TRBC1, CD3E, CD3D, GZMA, CD8A, CD2, GZMB, CD247, KLRC1, LAG3, CYTIP, ACTG2 |
| Endothelial cells_1 | VWF, EGFL7, CD34, CLEC14A, CD93, MMRN2, PECAM1, CAVIN1, ECSCR, RAMP2, ADGRL4, BTNL9, NPDC1, IL3RA, CAVIN2 |
| Endothelial cells_2 | CD34, VWF, CLEC14A, EGFL7, CD93, PECAM1, MMRN2, CAVIN1, ECSCR, RAMP2, ADGRL4, NPDC1, TCF4, ADAMTS1, IL3RA |
| M2 Macrophages | CD14, MPEG1, MS4A6A, CD163, KCNMA1, FCGR3A, LILRB2, MS4A4A, FGL2, PLA2G7, FCGR1A, CD4, CD68, IRF8, MRC1 |
| Macrophages | CD14, MPEG1, MS4A6A, FCGR3A, FGL2, FCGR1A, LILRB2, CD163, KCNMA1, MS4A4A, CD4, AIF1, PLA2G7, CD68, MRC1 |
| Myeloid cells | CD14, MPEG1, MS4A6A, SERPINB9, FCGR3A, FGL2, LILRB2, CD163, KCNMA1, FCGR1A, PLA2G7, MS4A4A, CD4, IRF8, PTPRC |
| Myeloid dendritic cells | SERPINB9, FGL2, SPI1, MPEG1, MS4A6A, IRF8, CD14, CD4, CSF2RA, AIF1, LILRB2, PTPRC, ACTG2, MNDA, HAVCR2 |
| Myofibroblasts_1 | COL5A2, PDGFRB, VCAN, THY1, PCOLCE, FBN1, ACTA2, THBS2, MYLK, CAVIN1, LTBP2, CRISPLD2, ASPN, FBLN1, CTSK |
| Myofibroblasts_2 | COL5A2, PDGFRB, VCAN, THY1, THBS2, FBN1, PCOLCE, LTBP2, MYLK, ACTA2, FBLN1, ASPN, PDGFRA, CRISPLD2, CAVIN1 |
| Plasma/B cells_1 | TENT5C, MZB1, PRDM1, FKBP11, CD79A, PLCG2, CYTIP, PECAM1, SLAMF7, DERL3, TNFRSF17, CD27, MEF2C, TCF4, SLAMF1 |
| Plasma/B cells_2 | TENT5C, MZB1, PRDM1, FKBP11, CD79A, PLCG2, CYTIP, PECAM1, SLAMF7, DERL3, TNFRSF17, CD27, MEF2C, TCF4, SLAMF1 |
| SERPINB9+ endothelial cells | CD34, VWF, EGFL7, CLEC14A, CD93, PECAM1, MMRN2, SERPINB9, CAVIN1, ECSCR, RAMP2, ADGRL4, NPDC1, TCF4, ADAMTS1 |
| Squamous CC_1 | TNC, COL17A1, CLCA2, GPC1, SLITRK6, LTBP2, CAVIN1, MYC, APCDD1, TACSTD2, FHL2, DST, EGFR, MET, SEMA3C |
| Squamous CC_2 | COL17A1, TNC, CLCA2, SLITRK6, GPC1, CAVIN1, LTBP2, MYC, DST, FBLN1, TACSTD2, FHL2, EGFR, MET, APCDD1 |
| Squamous CC_3 | CLCA2, TNC, COL17A1, GPC1, SLITRK6, TACSTD2, MYC, CAVIN1, LTBP2, EGFR, SERPINB9, FBLN1, MET, APCDD1, DST |
| Squamous CC_4 | TNC, COL17A1, LCA2, GPC1, LTBP2, APCDD1, SLITRK6, CAVIN1, MYC, FHL2, EGFR, DST, SEMA3C, TACSTD2, MET |
| Squamous CC_5 | TNC, COL17A1, CLCA2, SLITRK6, GPC1, LTBP2, CAVIN1, MYC, APCDD1, EGFR, TACSTD2, DST, FHL2, MET, FBLN1, |
