# Supplementary material for: Spatially resolved T cell receptor diversity mapping uncovers variability of the cancer immune microenvironment
Source: eBioMedicine. 2026 Apr 24;127:106264. doi: 10.1016/j.ebiom.2026.106264 (PMC13127328; doi:10.1016/j.ebiom.2026.106264)
Supplement: Supplementary Material- Methods [file mmc9.docx]

**Supplementary Methods**

**RNA sequencing**

RNA Sequencing data analyzed in this study were generated and published previously ^1,2^. No new RNA sequencing was performed in the present study. Transcriptomic profiling was conducted on fresh-frozen tumor tissue, as described previously ^2^. Briefly, RNA was extracted using the RNeasy Mini Kit (Qiagen) from archived fresh-frozen tumor tissues stored at −80°C. For each sample, five tissue sections of 10 μm thickness were used. Libraries were prepared using the Illumina TruSeq RNA Sample Prep Kit V.2 with poly-A selection. Paired-end multiplex sequencing (100 bp) was performed on the Illumina HiSeq2500 platform (Illumina, USA), with five samples per lane, following the standard Illumina RNAseq protocol. Raw sequencing data are publicly available in the Gene Expression Omnibus (GEO) repository (accession number GSE81089).

**Lymphotrack**

Sequencing data analyzed in this study were generated and published previously ^1^. No new LymphoTrack sequencing was performed in the present study. Raw LymphoTrack sequencing files are not publicly deposited for personal data protection regulations, but are available from the original authors upon reasonable request.

Briefly, the Lymphotrack assay was performed as described previously ^1,3^. Three 10 μm fresh-frozen tissue sections of each NSCLC patient sample were transferred into ATL buffer, and DNA was extracted using DNeasy Blood and Tissue kit (Qiagen Germany) according to the manufacturer’s protocol. Extracted DNA was eluted in 100 μL AE buffer, and the concentration was measured by the Qubit V.2.0 Fluorometer (Invitrogen) with the Qubit dsDNA BR Assay Kit (Invitrogen, USA) according to the manufacturer’s protocol. TRB and TRG rearrangements were assessed respectively using LymphoTrack Dx TRB Assay Kit A-MiSeq (Invivoscribe, USA) and LymphoTrack Dx TRG Assay Kit Panel-MiSeq (Invivoscribe) on the Illumina MiSeq sequencing platform. Raw sequence data was analyzed and visualized using the LymphoTrack Software-MiSeq (V.2.4.3, Invivoscribe).

**In situ sequencing**

In situ sequencing data analyzed in this study were generated and published previously by Yu et al., 2025 ^1^. A subset of the data was reused here for method comparison. In situ sequencing was performed as previously described by Lee et al. ^4,5^. Briefly, 10 µm frozen tumor sections were fixed, permeabilized, and processed for padlock probe hybridization and rolling circle amplification using the Cartana High Sensitivity Library Preparation and In Situ Sequencing kit, according to the manufacturer’s protocol. Libraries targeting immune- and TCR-related transcripts were generated, followed by iterative probe hybridization, imaging, and stripping cycles. Image acquisition and data processing were carried out as described previously ^4^. The data analysis code is available at <https://github.com/Moldia/Lee_2023>. No de novo sequencing data were generated via the in situ sequencing experiments, consequently no raw sequencing data or sequence accession numbers are applicable to these experiments.

**Spearman correlation**

The Spearman rank correlation coefficient (r_s_ ) was calculated using GraphPad Prism (10.6.1) to compare the detection of TRBV genes across the Xenium, Lymphotrack, RNAseq and In Situ Sequencing (ISS) datasets. The Spearman correlation was selected as the appropriate metric since it does not assume normality and is robust to outliers, compared to other commonly used parametric correlation measures ^6,7^.

**Probe selection and design**

The individual TR exon sequences were split in all possible k-mers, by applying a sliding window function with k=30. Each 30-mer was checked for matches against all the TCR transcript sequences of the same gene group, by utilizing the vmatchPattern function from the Biostrings R package, that checks a group of sequences for the presence of a pattern or query sequence. Imperfect matches with up to 6 mismatching residues (20% of the total length of a 30-mer) were also considered matches. In a first filtering step, we excluded 30-mers that had matches in more than 10 TCR transcript sequences of one gene group, as those ones would be highly unspecific targets for PLPs. The remaining 30-mers were filtered, in a second filtering step, for GC content between 40 and 60%, presence of G or C in position 16 (corresponds to probe 3΄ ligation site and is required by the RNA-based ISS chemistry) and absence of more than 4 repetitive nucleotides. The filtered 30-mers were manually selected so that, if possible, each unique TCR gene is targeted by 3 PLPs. Few TCR genes, due to very highly conserved sequence identity, were targeted as a group. For example, TRBC1 and TRBC2 are grouped and detected together (Fig. 1B, Sup. Table 3). The presence of the filtered 30-mers in other, non-specific human non-TCR sequences was checked against the human Transcriptome, using the vmatchPattern function. The final k-mers were used for the design of padlock probes with 10x designed backbone which includes the barcode sequence of each probe and the L-probe binding sequence (Sup. Table 4).

**Xenium In Situ Gene Expression and tissue processing**

The fresh frozen tissue was fixed and permeabilized as described in Xenium Fixation and Permeabilization Protocol (Demonstrated protocol CG000581). FFPE tissues were deparaffinized and permeabilized to make the mRNA accessible as described in Xenium Deparaffinization and Decrosslinking Protocol (Demonstrated protocol CG000580). Predesigned Human Multi-Tissue and Cancer panel (377 genes) and custom TCR gene panel (98 genes) were added to the tissue. Probes were hybridized to the target RNA, ligated, and enzymatically amplified generating multiple copies for each RNA target as described in Probe Hybridization, Ligation and Amplification user guide (User guide CG000582). The samples deriving from the PREDIX LumB cohort were additionally stained for cell segmentation according to the manufacturer’s protocol (Xenium In Situ Gene Expression with Cell Segmentation Staining, User Guide CG000749). Fluorescent markers included in the staining kit target nuclei (DAPI), membrane boundaries (ATP1A1, CD45, E-Cadherin), interior RNA (18S RNA), and interior proteins (αSMA, Vimentin).

**Label transfer for clustering annotations**

For the clustering and cell annotation of samples deriving from the PREDIX LumB cohort, we performed label transfer from the annotated scRNA-seq dataset ^8^ onto our Xenium spatial data using Scanpy (version 1.10.4) in Python (version 3.11.3) via its 'ingest' functionality. First, both reference and query (Xenium) datasets were restricted to their shared gene set, normalized (e.g. total-counts normalization + log1p), and the reference data was projected via PCA and nearest-neighbor graph inference. After UMAP embedding and clustering of the reference, we ran sc.tl.ingest(query, reference, obs='cell_type') to map the query cells into the reference embedding and assign the most likely cell type labels. Low-confidence or ambiguous assignments were flagged or left as “unassigned,” and we validated transferred annotations by checking canonical marker gene expression in the spatial data.

**Neighborhood analysis using Squidpy**

Spatial proximity and neighborhood enrichment were assessed using permutation-based tests on spatial neighbor graphs constructed with Squidpy ^9^ (<https://github.com/scverse/squidpy>) by selecting the neighboring cells within a 125-pixel radius for each cell, which approximately corresponds to a two-cell diameter.

As described before ^9^, a spatial graph was created where cells are identified as nodes and neighborhood relationships between cells as edges (spatial connectivities). Then the association between label pairs (cell types or TCRVβ/Vα pairs) in the connectivity graph is estimated by counting the sum of nodes that belong to classes i and j and are proximal to each other, noted xij. Permutation testing was performed by repeatedly scrambling cell labels across the fixed spatial connectivity graph (1,000 times by default) and recalculating neighbor interaction counts to estimate the null expectation. Expected means (μ_ij_), standard deviations (σ_ij_) and a Z-scores for each pair were calculated.

**Gini Index**

The Gini Index (Gini coefficient) was calculated to assess the inequality in TRV transcript counts among cells in clusters. The analysis was performed using the AnnData object, containing Xenium data after cell segmentation in the form of single-cell RNA sequencing data. Specifically, genes starting with 'TRBV' or 'TRAV' were filtered from the dataset. Clusters that had been identified as BANKSY Spatial domain 2 with more than 50 cells, were identified using the dbscan_clusters column in the AnnData object. For each cluster, transcript counts were extracted and filtered to remove zero values. The Gini coefficient was then calculated using the following formula:

$$G=1-2\left( \frac{\sum_{i=1}^{n+1} F_{i}-\frac{F_{n+1}}{2}}{F_{n+1}\cdot n} \right)$$

where $F_{i}$represents the cumulative sum of sorted fractions (including 0 at the beginning), n is the total number of original fraction (n+1 include the added 0), $F_{n+1}$ is the total sum of the sorted cumulative fractions (last cumulative value).

**Jaccard Similarity Index**

The abundance-based Jaccard Similarity Index (J_abd_) was calculated to highlight the overlap of TCRVβ/Vα pairs in biopsies from patients with breast cancer between two time points (baseline and visit). J_abd_ weights shared TCRVβ/Vα pairs by their relative contribution to the repertoire of each timepoint and unlike incidence-only based indices, is suitable for assessing compositional similarity between unequal-sized samples or samples likely to contain numerous rare species.

The J_abd_ was calculated based using the following formula as described by Chao et al. ^10^ :

$$Ĵabd=\frac{ÛV}{Û+V - ÛV}$$

U and V represent the total abundances of the shared species in time point 1 and 2, respectively. Both indices reach 1 for identical assemblages and tend to 0 for disjoint assemblages

$$Û=\sum_{i=1}^{D\text{12}} \frac{X\text{i}}{n}+\frac{(m-1)}{m} \frac{f\text{+1}}{2f\text{+2}}\sum_{i=1}^{D\text{12}} \frac{X\text{i}}{n}I(Y\text{i}=1)$$

$$V=\sum_{i=1}^{D\text{12}} \frac{Y\text{i}}{m}+\frac{(n-1)}{n} \frac{f\text{1+}}{2f\text{2+}}\sum_{i=1}^{D\text{12}} \frac{Y\text{i}}{m}I(X\text{i}=1)$$

**References**

1. Yu H, Magoulopoulou A, Amini RM, et al. Spatial TCR clonality and clonal expansion in the in situ microenvironment of non-small cell lung cancer. *J Immunother Cancer*. 2025;13(8):e012089. doi:10.1136/jitc-2025-012089

2. Djureinovic D, Hallström BM, Horie M, et al. Profiling cancer testis antigens in non–small-cell lung cancer. *JCI Insight*. 2016;1(10). doi:10.1172/jci.insight.86837

3. Paulsen K, Marincevic M, Cavelier L, Hollander P, Amini RM. LymphoTrack Is Equally Sensitive as PCR GeneScan and Sanger Sequencing for Detection of Clonal Rearrangements in ALL Patients. *Diagnostics (Basel)*. 2022;12(6):1389. doi:10.3390/diagnostics12061389

4. Lee H, Langseth CM, Salas SM, et al. Open-source, high-throughput targeted in situ transcriptomics for developmental and tissue biology. *Development*. 2024;151(16):dev202448. doi:10.1242/dev.202448

5. Lee H, Marco Salas S, Gyllborg D, Nilsson M. Direct RNA targeted in situ sequencing for transcriptomic profiling in tissue. *Sci Rep*. 2022;12(1):1. doi:10.1038/s41598-022-11534-9

6. Huang AL, He YZ, Yang Y, Pang M, Zheng GP, Wang HL. Exploring the potential of the TCR repertoire as a tumor biomarker (Review). *Oncol Lett*. 2024;28(3):413. doi:10.3892/ol.2024.14546

7. Reuben A, Zhang J, Chiou SH, et al. Comprehensive T cell repertoire characterization of non-small cell lung cancer. *Nat Commun*. 2020;11(1):603. doi:10.1038/s41467-019-14273-0

8. Wu SZ, Al-Eryani G, Roden DL, et al. A single-cell and spatially resolved atlas of human breast cancers. *Nat Genet*. 2021;53(9):1334-1347. doi:10.1038/s41588-021-00911-1

9. Palla G, Spitzer H, Klein M, et al. Squidpy: a scalable framework for spatial omics analysis. *Nat Methods*. 2022;19(2):2. doi:10.1038/s41592-021-01358-2

10. Chao A, Chazdon RL, Colwell RK, Shen TJ. A new statistical approach for assessing similarity of species composition with incidence and abundance data. *Ecology Letters*. 2005;8(2):148-159. doi:10.1111/j.1461-0248.2004.00707.x
